# A Review of Exotic Animal Disease in Great Britain and in Scotland Specifically between 1938 and 2007

**DOI:** 10.1371/journal.pone.0022066

**Published:** 2011-07-27

**Authors:** Onneile O. Peiso, Barend M. de C. Bronsvoort, Ian G. Handel, Victoriya V. Volkova

**Affiliations:** 1 Epidemiology Group, School of Biological Sciences, Centre for Infectious Diseases, University of Edinburgh, Edinburgh, United Kingdom; 2 Epidemiology, Economics and Risk Assessment (EERA) Group, The Roslin Institute and Royal (Dick) School of Veterinary Studies, University of Edinburgh, Easter Bush, Roslin, Midlothian, United Kingdom; University of Hong Kong, Hong Kong

## Abstract

**Background:**

Incursions of contagious diseases of livestock into disease-free zones are inevitable as long as the diseases persist elsewhere in the world. Knowledge of where, when and how incursions have occurred helps assess the risks, and regionalize preventative and reactive measures.

**Methodology:**

Based on reports of British governmental veterinary services, we review occurrence of the former OIE List A diseases, and of Aujeszky's disease, anthrax and bovine tuberculosis (bTB) in farm-animals in Great Britain (GB) between 1938 and 2007. We estimate incidence of each disease on GB agricultural holdings and fraction of susceptible farm-animals culled to control the disease each year. We then consider the frequency and incidence of the diseases in Scotland alone. The limitations of available data on historical disease occurrence and denominator populations are detailed in Text S2.

**Conclusions:**

The numbers of livestock and poultry farmed in GB grew over the years 1938–2007; the number of agricultural holdings decreased. An amalgamation of production on larger holdings took place from the 1940s to the 1980s. The maximum annual incidence of a reviewed disease in GB 1938–2007 was reported for bTB, 1.69% of holdings in 1961. This was followed by Newcastle disease, 1.50% of holdings in 1971, and classical swine fever, 1.09% of holdings in 1940. The largest fractional cull of susceptible livestock in a single year in each of the four decades 1950s–1980s was due to a viral disease primarily affecting swine. During the periods 1938–1949 and 2000–2007 this was due to outbreaks of foot and mouth disease. In the absence of incursions of the former OIE List A diseases in the 1990s, this was due to bTB. Over the 70 years, the diseases were reported with lower frequency and lower annual incidence in Scotland, as compared to when these statistics are considered for GB as a whole.

## Introduction

The introduction of exotic disease into an immunologically naïve farm-animal population has potentially devastating consequences for animal welfare, animal production and the wider economy. In the first decade of the 21st century, Great Britain (GB) has seen several major disease introductions, including classic swine fever (CSF) in 2000 [1], foot and mouth disease (FMD) in 2001 [2]–[4], and bluetongue in 2008 [5]. The expansion of long-distance livestock movements across Europe, and the massive expansion of wider trade in animals and animal products make Britain increasingly vulnerable to unwanted disease intrusions. This raises unresolved tensions between the various stake-holders as to how much control should be imposed by governments, and who should pay the costs of control and compensation. Changes in demographics of livestock within Britain also impact on the potential for infections to spread.

Much however can be done to minimize the frequency of exotic disease incursions and to improve the control of subsequent outbreaks through understanding disease ecology and human behaviour. Knowledge of where, when and how the incursions have occurred and progressed is critical for assessment of future risks, choice of preventive measures and decision-making (*e.g.* regionalization) during disease outbreaks. Here we review the occurrence of the former OIE List A diseases, Aujeszky's disease, anthrax and bovine tuberculosis (bTB) in GB and in Scotland particularly, during the 70 years between 1938 and 2007. Aujeszky's disease, anthrax and bTB were included in the review following a consultation with the Animal Health and Welfare Division of the Scottish Government, due to the major economic consequences of their occurrence. The former OIE List A included: African horse sickness, African swine fever, highly-pathogenic avian influenza (HPAI), bluetongue, classical swine fever (CSF), contagious bovine pleuropneumonia, exotic Newcastle disease (NDV), foot and mouth disease (FMD), goat and sheep pox, lumpy skin disease, peste des petits ruminants, Rift Valley fever, rinderpest, swine vesicular disease (SVD) and vesicular stomatitis.

## Methods

### Data

The primary source of data on disease occurrence was the reports of British governmental veterinary services [6]. These reports evolved over the review period: following World War II, a single edition Report of Proceedings under the Disease of Animals Acts was compiled for the years 1938 to 1947; reports on the Animal Health Services in Great Britain were produced from 1948 to 1970; and the Annual Report of Chief Veterinary Officer, Animal Health was published from 1971 to 2007. We cross-checked the numbers of holdings affected by each disease in GB each year between 1938 and 2007 indicated in the veterinary services' reports against the outbreak statistics published on-line by the UK Government's Department for Environment, Food and Rural Affairs (DEFRA) (except for bTB where the latter has only been available since 1998), and from 1996 onwards also against the OIE HANDISTATUS II published on-line (http://www.oie.int/hs2/report.asp). Contagious diseases of farmed fish were not reviewed because a source of consistent historical data was not identified.

The database on disease occurrence was developed following the recommendations of the Preferred Reporting Items for Systematic Reviews and Meta-Analyses (PRISMA) statement [7]. The details are provided in *Text S1. Conduct of the systematic review*. In short, a searchable database was compiled in Microsoft Office Access ®2003 using a specifically-designed data extraction form. For each disease in each year reviewed, for both GB and Scotland specifically, the form included: total number of holdings affected and animals culled, mode(s) of introduction, mode(s) of the subsequent spread, animal species with which the disease was introduced and spread, all animal species affected, number of index holdings, and the localities of index holdings and of all the holdings affected. The duration of outbreaks was not recorded because some of the diseases reviewed were endemic in GB during a part of the review period.

Information on the denominator farm-animal populations in GB between 1938 and 2007 was collated. The primary source of these data was the results of the annual agricultural censuses conducted in all three countries of GB.

Data sources, data availability and limitations are detailed in *Text S2. Data sources, availability and limitations*.

### Disease indexes

For each disease in each year reviewed, two indexes were calculated:

1. Cumulative annual incidence, estimated as the percentage of total agricultural holdings affected by the disease in GB, and in Scotland specifically.

We use the term cumulative ‘annual incidence’ for the index (1) rather than period-prevalence. In the beginning of the review period some of the diseases reviewed were endemic in parts of GB on the regional level. However, throughout the review period animals detected as having a reviewed disease were culled if they had not died, and every effort was made to eliminate the disease on the premises. We therefore assume that the vast majority of the holdings reported as being affected in a given year were new cases of disease. In the later part of the review period, all holdings reported as affected by exotics were new cases.

1. The percentage of susceptible farm-animals culled due to the disease in GB. This included all farm-animals of susceptible species that were slaughtered. For the viral diseases this was comprised of the animals diseased and culled for control purposes; for anthrax by the diseased animals; and for bTB by the animals detected as being infected and therefore assumed to be subsequently slaughtered. The species counted as susceptible were: pigs for Aujeszky's disease, CSF and SVD; cattle for bTB; cattle, sheep and pigs for anthrax and FMD; and all farmed poultry for NDV and HPAI.

Additionally, the percentages of holdings farming susceptible animal species affected by the disease in GB and in Scotland in each year reviewed were estimated for Aujeszky's disease, CSF, SVD, bTB and NDV. However, these statistics should be interpreted with caution due to limitations of the denominator data (see *Text S2. Data sources, availability and limitations*).

## Results

Of the former OIE List A diseases, CSF, FMD, SVD, HPAI (“fowl plague” until 1981 [8]) and NDV (“fowl pest” until 1962) have all occurred in GB between 1938 and 2007. The remaining OIE List A diseases did not occur: African horse sickness, African swine fever, bluetongue (introduced in 2008), contagious bovine pleuropneumonia, goat and sheep pox, lumpy skin disease, peste des petits ruminants, Rift Valley fever, rinderpest and vesicular stomatitis.

### Livestock demographics in Great Britain 1938–2007

**Figure 1:**
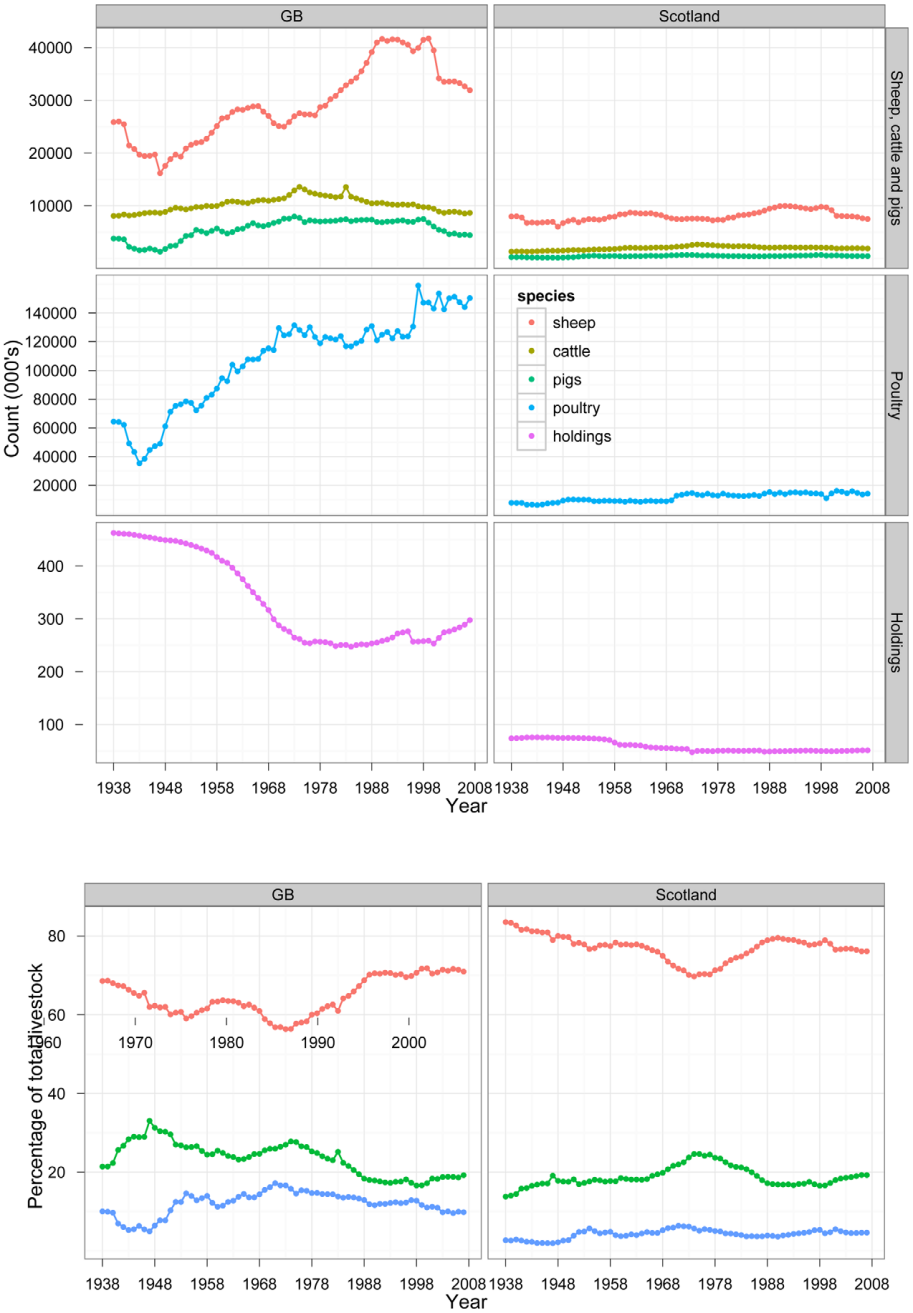
Farm-animal demographics and the numbers of agricultural holdings each year 1938–2007 in Great Britain (left panels) and in Scotland (right panels).

The total number of cattle, sheep and pigs farmed in GB almost doubled from 32.7 million in the 1940s to 58.2 million in the 1990s, but then declined following the FMD outbreak in 2001 to 46.5 million (Figure 1, left panels; see *Table S1* for all 70-year results). The number of poultry more than doubled from 61.1 million in 1948 to 150.4 million in 2007 (Figure 1, left panels; *Table S1*). Conversely, the number of agricultural holdings registered in GB decreased from 460,000 in the late 1930s to 250,000 in the 1980s (Figure 1, left panels; *Table S1*). This reflected the amalgamation of agricultural production onto larger holdings between the 1940s and the 1980s. The number of holdings subsequently grew to around 300,000 in 2007. The species-composition of the livestock farmed in GB had remained relatively stable over the years 1938–2007 (Figure 1, left panels).

### Individual diseases in Great Britain 1938–2007

**Figure 2:**
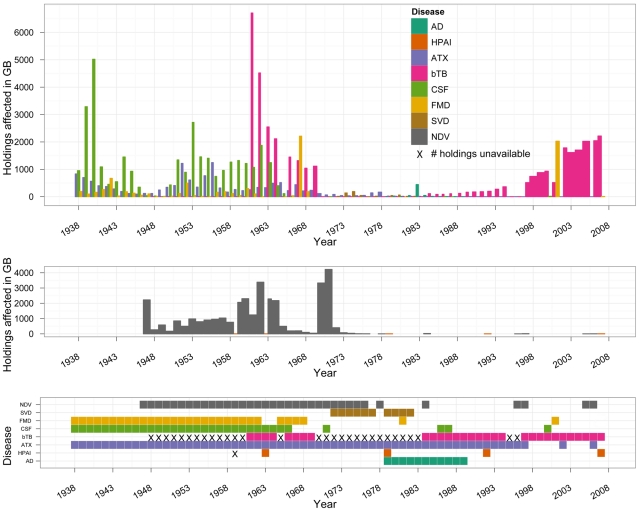
Disease frequency and numbers of holdings affected by reviewed diseases in Great Britain 1938–2007. The numbers of herd breakdowns with bTB in GB were included in the reports of British governmental veterinary services since 1948, and the numbers of NDV outbreaks since 1947. AD - Aujeszky's disease, ATX-anthrax.

The occurrence of the reviewed diseases in GB from 1938 to 2007 and the numbers of holdings affected are summarized in Figure 2. The modes of disease introduction (sources of disease) in GB and into Scotland specifically, and the modes of subsequent disease spread within GB and within Scotland were noted whenever available in the reports of British governmental veterinary services. The availability was limited, and the modes mentioned at least once are summarized below. Affected animal species reported are also included.

### FMD

**Figure 3:**
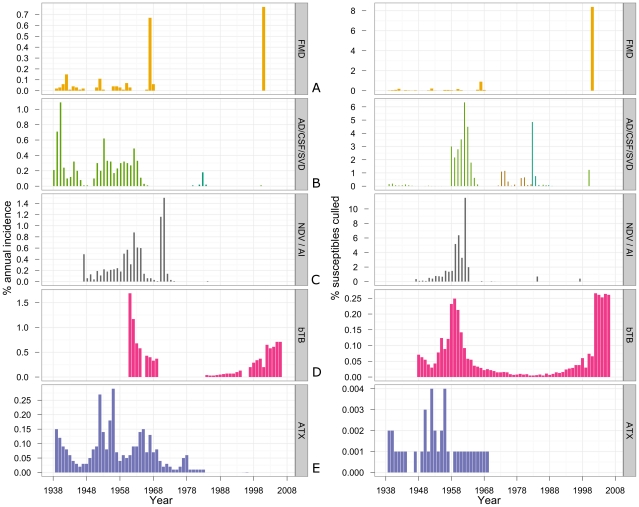
Annual incidence on agricultural holdings (left panels) and fractional cull of susceptible farm-animals (right panels) in Great Britain 1938–2007 for: (A) foot and mouth disease; (B) Aujeszky's disease, classical swine fever and swine vesicular disease; (C) Newcastle disease and HPAI; (D) bovine tuberculosis; and (E) anthrax. The numbers of herd breakdowns with bTB in GB were included in the reports of British governmental veterinary services since 1948, and the numbers of NDV outbreaks since 1947. AD - Aujeszky's disease, ATX-anthrax. The numbers of poultry culled in GB due to NDV were not available for 8 of 36 years the disease was reported: 1965, 1966, 1972–1976 and 2006. The numbers of poultry culled due to HPAI were not available for 3 of 5 years reported: 1963, 2006 and 2007. The numbers of animals culled due to Aujeszky's disease were not available for 4 of 11 years reported: 1979–1982.

Foot and mouth disease has been recorded in GB since at least 1839; it has been notifiable since 1869. In the 70 years from 1938 to 2007, FMD occurred in GB in a total of 32 years (Figure 2, bottom panel). However, the frequency of disease occurrence changed over the review period. Outbreaks of FMD occurred every year between 1938 and 1962; but only in 7 years between 1963 and 2007, of which 4 years were before 1969. Also, the FMD outbreak in 2007 was due to a technological failure rather than an introduction (and therefore is not included in Figure 2). The highest number of holdings affected by FMD was 2,210 (0.67% of total GB agricultural holdings) in 1967, followed by 2,026 (0.77%) in 2001 (Figure 2, top panel). The maximum annual incidence of FMD in GB over the 70 years was 0.77% of holdings in 2001 (Table 1; Figure 3, left panels). The 2001 outbreak also had the highest risk of slaughter per susceptible animal farmed, with 8.38% of cattle, sheep and pigs farmed in GB that year culled for control (Table 2; Figure 3, right panels). Prior to 2001, the largest fraction of cattle, sheep and pigs culled in control of FMD in a single year was 0.90% in 1967 (Figure 3, right panels). Considering the FMD 2001 fractional cull by species, 6.66% of cattle, 9.69% of sheep and 2.61% of pigs farmed in GB that year were culled. Of the total cull, 0.05% was comprised by other animals. Of the cattle, sheep and pigs culled during the FMD 2001 outbreak, 31.89% were on holdings known to be infected, 30.56% were on dangerous contact (DC) contiguous premises, and 37.30% were on DC non-contiguous premises including those within 3 km radius of affected holdings (calculated using the nominator data from Anderson (2002) [9]). Translating this into the fractional cull of the total cattle, sheep and pigs farmed that year (note rounding errors), 2.66% were culled on infected premises, 2.55% on DC contiguous premises, and 3.11% on DC non-contiguous premises.

**Table 1:** Maximal annual incidence of reviewed diseases on agricultural holdings of Great Britain in 1938–2007^a^.

disease	year of maximalannual incidence	annual incidence, %
bTB	1961	1.69
NDV	1971	1.50
CSF	1940	1.09
FMD	2001	0.77
anthrax	1956	0.29
Aujeszky's disease	1983	0.18
SVD	1974	0.07

a HPAI is not included due to its negligible incidence.

**Table 2:** Maximal annual cull of farm-animals in Great Britain due to each disease reviewed 1938–2007^a^.

disease	year of maximal annual cull	susceptibles counted	susceptibles culled, %
NDV	1962	all poultry	11.53
FMD	2001	cattle, sheep, pigs	8.34
CSF	1962	pigs	6.34
Aujeszky's disease	1983	pigs	4.86
SVD	1974	pigs	1.16
bTB	2002, 2007	cattle	0.27
anthrax	1952, 1956	cattle, sheep, pigs	0.004

a HPAI is not included due to its negligible incidence.

Notably, the annual incidence of FMD in GB prior to the first-time eradication of the disease in the 1960s showed a cyclicity with a peak every 10 years, and an occasional extremely large peak as in 1967–1968. This was similar to the approximately once in 10 years peak (with an occasional extreme peak) in the numbers of holdings affected with FMD and of diseased animals observed in Germany between 1886 and 1925 [10]. In both cases, no vaccination against FMD was practiced. It is hard to speculate on what was driving those cyclical patterns.

The modes of FMD introduction included: imported contaminated meat; imported meat wrappers; contaminated animal carcasses; contaminated meat, meat-scraps and bones; swill; infected animals; airborne with wind; by wild birds; and a leak from research facilities due to a failure of biosecurity. Subsequent spread from incursions was reported to occur via swill; due to sale of infected animals via markets; due to pigs being allowed to range in the forest; by people, including on footpaths; by hedgehogs; by migratory birds; on contaminated lorries; “mechanical”; and in contaminated milk. Transmission in markets was reported. Cattle, sheep, pigs, goats, “other cloven-hoofed animals” and hedgehogs were reported to be affected by FMD.

### Swine exotics

CSF was confirmed in GB for the first time in 1864; the first measures to control this disease were introduced in 1878. In total, CSF was reported in GB in 33 years of the 70 years from 1938 to 2007, but mainly prior to 1966 (Figure 2, bottom panel). The maximum incidence of CSF was observed in 1940, with outbreaks on 1.09% of GB agricultural holdings (Table 1; Figure 3, left panels). However, the highest fractional cull of pigs farmed in GB in control of CSF was 6.34% in 1962, followed by 4.49% in 1963 (Table 2; Figure 3, right panels). CSF was eradicated in GB for the first time in 1966; after that, only outbreaks of a limited size (at most 16 holdings affected) occurred: in 1971, 1986 and 2000 (Figure 2, top panel).

SVD occurred in GB in 10 years between 1972 and 1982 (Figure 2, bottom panel). Its maximum incidence on GB holdings was 0.07% in 1974 (Table 1; Figure 3, left panels). In control of SVD, 1.10% of pigs farmed in GB were culled in 1973, followed by the maximum for this disease - 1.16% of those farmed in 1974 (Table 2; Figure 3, right panels).

Aujeszky's disease was reported in GB in 11 years between 1979 and 1991 (Figure 2, bottom panel). Its maximum incidence on GB holdings was 0.18% in 1983 (Table 1; Figure 3, left panels). In the same year, there was the maximum annual fractional cull of pigs farmed in GB in control of Aujeszky's disease, 4.86%, (Table 2; Figure 3, right panels).

Therefore, the maximal cull in control of an exotic viral disease of swine occurred several years before eradication of the disease from the GB farm-pig population. There was a period of several years between the maximal cull and the eradication of the disease.

In terms of the modes of introduction and transmission, the introduction of CSF was recorded as having occurred via infected animals, swill, and transmission in livestock markets and in transit. Recrudescence of the disease was also reported. The subsequent spread of the disease was reported as occurring through: neighbourhood spread; with purchase/usage of infected breeding pigs; by mechanical means (on clothing, utensils and other); and by people. Transmission in livestock markets, during transportation and in transit was reported.

For SVD, swill was the only mode of introduction mentioned. The subsequent spread was reported as occurring through: sale of infected animals via markets or farm-to-farm; mechanical transmission on contaminated lorries and “vehicles”; neighbourhood spread; people, via movement of personnel; and with contaminated food waste and bakery waste. In addition to pigs, it was reported that cattle, sheep and goats were also affected; but these non-swine species were reported only in 1972 - the first year when the disease was registered in GB.

For Aujeszky's disease, the movement of pigs was reported as the mode of introduction. Among the farm animals, only pigs were mentioned as being affected.

### Avian exotics

Outbreaks of Newcastle disease have been reported in poultry in GB since the 1930s (although the disease may have been present earlier); the numbers of affected holdings have been included in the reports of British governmental veterinary services since 1947. The disease was reported in GB in 36 of the 61 years between 1947 and 2007 (Figure 2, bottom panel). However, only outbreaks of a limited size (at most 35 holdings affected) occurred after 1973 (Figure 2, middle panel). The maximum incidence of NDV on GB agricultural holdings was 1.50%, recorded in 1971 (Table 1; Figure 3, left panels). But the maximum losses of poultry due to NDV were encountered a decade earlier: 11.53% of poultry farmed in 1962 (Table 2; Figure 3, right panels). Licensing of vaccines with new viral strains in 1970 and 1971 was one of the factors that changed the relationship between the incidence of NDV and the losses of poultry [11]. The method of vaccine delivery also progressed to via-spray administration [12]. Nonetheless, the outbreaks of NDV in GB in 1970–1971 were estimated as having cost in excess of £20 million, which at the time was comparable to the losses from a serious epizootic of FMD [13].

The reported modes of NDV introduction included: illegal movement of poultry; purchase of psittacines; purchase of turkeys; swill; import of contaminated animal carcasses; and the sale of infected poultry via markets. As the modes of subsequent spread, the movement of poultry and neighbourhood spread throughout high-density poultry populations were mentioned. The poultry affected included broilers, ducks, geese, turkeys, pheasants and “others”.

Disease showing the symptom-complex of avian influenza (“fowl plague”) was described in farmed, backyard and free-living poultry in GB long before the concept of HPAI was defined in 1981 [8]. However, the occurrence of HPAI in poultry farmed in GB between 1938 and 2007 was limited to five episodes: in 1959 (H5N1), 1967 (H7N3), 1979 (H7N7), 1991 (H5N1), and 2007 (H5N1) (Figure 2, middle and bottom panels). The outbreak in 1959 involved chickens, while in the other four cases it involved turkeys.

### Bovine tuberculosis

The control programme for bTB in GB, based on comparative intradermal tuberculin sensitivity testing and slaughter of reactors began in 1947, and was made compulsory in 1950 [14]. The numbers of herd breakdowns (one or more positive reactors in a previously clean herd) with bTB and of cattle affected have been included in the reports of British governmental veterinary services since 1948. However, the numbers of breakdowns were not available in the reports for 30 of the 60 years 1948–2007 (Figure 2, bottom panel), although the numbers of cattle-reactors were available consistently.

Several inquiries into the epidemiology of bTB in GB were commissioned during the review period; we considered using the inquiries' reports as an additional source of the incidence information. However we concluded that for consistency with the other diseases reviewed only the bTB statistics in the reports of British governmental veterinary services would be used in this review. Acknowledging that there have been changes in the format of reporting bTB throughout the years, we note that the only bTB statistics we consider are the number of reactors to the comparative intradermal tuberculin sensitivity test and the number of affected holdings each year after the test-and-cull programme and the case notification in GB became compulsory.

The fractions of GB cattle diagnosed with bTB in the years 2002–2007 exceeded those during the 1950s: a rate of 0.25%–0.27% of cattle affected was recorded each year from 2002 to 2007, compared to 0.21%–0.25% each year from 1958 to 1960 (Figure 3, right panels). The overall annual maximum over the 60 years 1948–2007 was 0.27% of cattle affected, observed in both 2002 and 2007. The holding-level incidence of bTB in GB in 2007 was 0.74% (Figure 3, left panels). This was close to that of FMD in 2001, which was 0.77% (Figure 3, left panels).

Import of livestock was reported as the mode of bTB introduction. As the modes of subsequent spread, infected cattle and badgers, and sale of infected animals via markets were mentioned. Of animals farmed in GB, only cattle were reported to be affected by bTB.

### Anthrax

Episodes of anthrax were reported in GB in 62 of the 70 years 1938–2007 (Figure 2, bottom panel). The maximum annual incidence was recorded in 1956, with disease episodes on 0.29% of GB agricultural holdings (Table 1; Figure 3, left panels). The maximal fraction of cattle, sheep and pigs farmed in GB affected by anthrax in a given year was 0.004%, recorded in 1952 and 1956 (Table 2; Figure 3, right panels). Data describing the location of the affected premises were rarely available, preventing an evaluation of anthrax incidence by latitude. The dependency of anthrax incidence on latitude has been observed, for example, in the European part of Russia [10] and other parts of Eurasia [15]. An understanding of this dependency is necessary to predict the potential change in risk with projected local changes in climate [15].

For anthrax, the following sources of infection were reported: imported contaminated feedstuffs; contaminated feedstuffs, pasture, silage, fertilizer, water, mill waste, hide salt, leather-dust, meat (including that from knacker-yards), bone meal, wool washing effluent and wool shady, sewage sludge, manure and tannery waste. Recrudescence of the disease on the premises (due to contaminated soil) was reported. The species reported to be affected by anthrax were: cattle, pigs, sheep, horses, dogs, cats, mink, raccoon, cheetah and lion.

### Comparing the frequency and incidence of individual diseases in Great Britain 1938–2007

The frequency of occurrence of FMD and CSF in GB ultimately dropped after the 1960s, and that of NDV after 1973 (Figure 2). The successes in control of FMD, CSF and NDV were achieved despite the great rise in the numbers of livestock and poultry farmed (Figure 1, left panels). The first-time eradication of “old enemies” was followed by the emergence of several contagious livestock diseases (primarily those of swine) new to GB, some of which however were conquered relatively quickly (Figure 2, top and bottom panels). SVD was introduced in 1972 and eradicated in 1982. Aujeszky's disease was introduced in 1979 and eradicated by 1991. Conversely, porcine reproductive and respiratory syndrome, which is not addressed in this review, appeared in GB from no apparent source in 1991 [16], [17] to become endemic [17], [18].

Based on the available evidence, of the reviewed diseases bTB affected the largest number of holdings in GB in a single year between 1938 and 2007, with new breakdowns in 6,707 herds in 1961 (followed by 4,520 breakdowns in 1962) (Figure 2, top panel). It was followed by the diseases of the former OIE List A: CSF with 5,019 outbreaks in 1940; and NDV with 4,217 outbreaks in 1971 (Figure 2, top panel).

The maximum annual incidence (percentage of GB agricultural holdings affected in a given year) of a reviewed disease was observed for bTB which affected 1.69% of holdings in 1961 (Table 1; Figure 3, left panels; see also *Table S3* for all 70-year results). In this index, it was followed by NDV with 1.50% of holdings affected in 1971, and then CSF with 1.09% of holdings affected in 1940 (Table 1; Figure 3, left panels). The maximum cumulative annual incidence of FMD was 0.77% in 2001, of anthrax 0.29% in 1956, of Aujeszky's disease 0.18% in 1983, and of SVD 0.07% in 1974 (Table 1; Figure 3, left panels); the incidence of HPAI was negligible. The diseases with maximal incidence on GB agricultural holdings in a given year of each decade reviewed are outlined in Table 3.

**Table 3:** Reviewed disease with maximal annual incidence on agricultural holdings of Great Britain in each decade 1938–2007.

period	disease with maximal annual incidence	year of maximal annual incidence	annual incidence, %
1938–1949	CSF	1940	1.09
1950s	CSF	1953	0.62
1960s	bTB	1961	1.69
1970s	NDV	1971	1.50
1980s	Aujeszky's disease	1983	0.18
1990s	bTB	1999	0.34
2000–2007	FMD	2001	0.77

The information on the location of affected holdings within GB was available in a very limited number of cases (see *Text S2. Data sources, availability and limitations*), and therefore is not presented.

### Comparing the cull of susceptible farm-animals due to individual diseases in Great Britain 1938–2007

Between 1938 and 2007, the maximal annual fractional cull of susceptible farm-animals in GB was observed for NDV, with loss of 11.53% of poultry in 1962 (Table 2; Figure 3, right panels; see also *Table S4* for all 70-year results). The largest fraction of susceptible livestock culled in GB in control of a single disease in a single year was 8.38% of total cattle, sheep and pigs farmed culled due to FMD in 2001 (Table 2; Figure 3, right panels; see also *Table S5* for all 70-year results). Next in the fractional cull of livestock was CSF with 6.34% of pigs farmed culled for control in 1962.

Considered by decade, viral diseases primarily affecting swine (Aujeszky's disease, CSF and SVD) led to culling the largest fractions of susceptible livestock in GB in a single year in each of the following decades: the 1950s, 1960s, 1970s and 1980s (Table 4; see also *Table S5* for all 70-year results). However, the significance of individual swine diseases differed between the decades: CSF played a major role in the 1950s and the 1960s, followed by the emerging SVD in the 1970s, and Aujeszky's disease in the 1980s (Table 4; Figure 3, right panelss). During the periods 1938–1949 and 2000–2007 the outbreaks of FMD led to culling the largest fractions of susceptible livestock (Table 4). In the absence of the former OIE List A diseases in the 1990s, bTB was responsible for culling of the largest fraction of susceptible livestock in a given year (Table 4).

**Table 4:** Livestock disease reviewed with maximal annual cull of susceptibles farmed in Great Britain in each decade 1938–2007.

period	disease	year of maximal annual cull	susceptibles counted	susceptibles culled, %
1938–1949	FMD	1942	cattle, sheep, pigs	0.19
1950s	CSF	1958	pigs	3.00
1960s	CSF	1962	pigs	6.34
1970s	SVD	1974	pigs	1.16
1980s	Aujeszky's disease	1983	pigs	4.86
1990s	bTB	1998	cattle	0.06
2000–2007	FMD	2001	cattle, sheep, pigs	8.34

The maximum total fractional cull of livestock to control reviewed diseases in GB in a single year between 1938 and 2007 was 8.45%, recorded in 2001 (see *Table S6* for all 70-year results). In that year, 8.38% of total cattle, sheep and pigs farmed were culled in control of FMD, and an additional 0.07% of cattle were culled in control of bTB. Second to this was 6.44% of total livestock culled in 1962: 6.34% of pigs due to CSF; 0.09% of cattle due to bTB; and a total of 0.003% of cattle, sheep and pigs due to FMD and anthrax (*Table S6*). However in 1962, in addition to the livestock, 11.53% of poultry farmed were culled in control of NDV, making 1962 the year of the largest fractional cull of farm-animals as a whole (17.97%) in GB due to exotic diseases over the 70 years reviewed. The second largest total annual cull of farm-animals, 9.55%, was experienced just two years before, in 1960; again, this was primarily due to CSF and NDV, and less due to FMD and bTB.

### Livestock demographics in Scotland 1938–2007

The number of livestock farmed in Scotland was growing more modestly compared to the rest of GB, increasing from 8.7 million of cattle, sheep and pigs in the 1940s to 12.3 million in the 1990s (Figure 1, right panels; see *Table S2* for all 70-year results). This was followed by a decline to 10.3 million after the FMD 2001 outbreak. The species-composition of livestock in Scotland had been incredibly stable, with sheep constituting 76%–82% of the total headage in any given year between 1938 and 2007, with the exception of the 1970s (Figure 1, right panels). The number of poultry, with some fluctuations, doubled from the 1940s to the 2000s (Figure 1, right panels). The number of agricultural holdings registered in Scotland decreased during the late 1950s and the 1960s, but at a steeper rate than in the rest of GB (Figure 1, right panels).

### Disease frequency and incidence in Scotland 1938–2007

**Figure 4:**
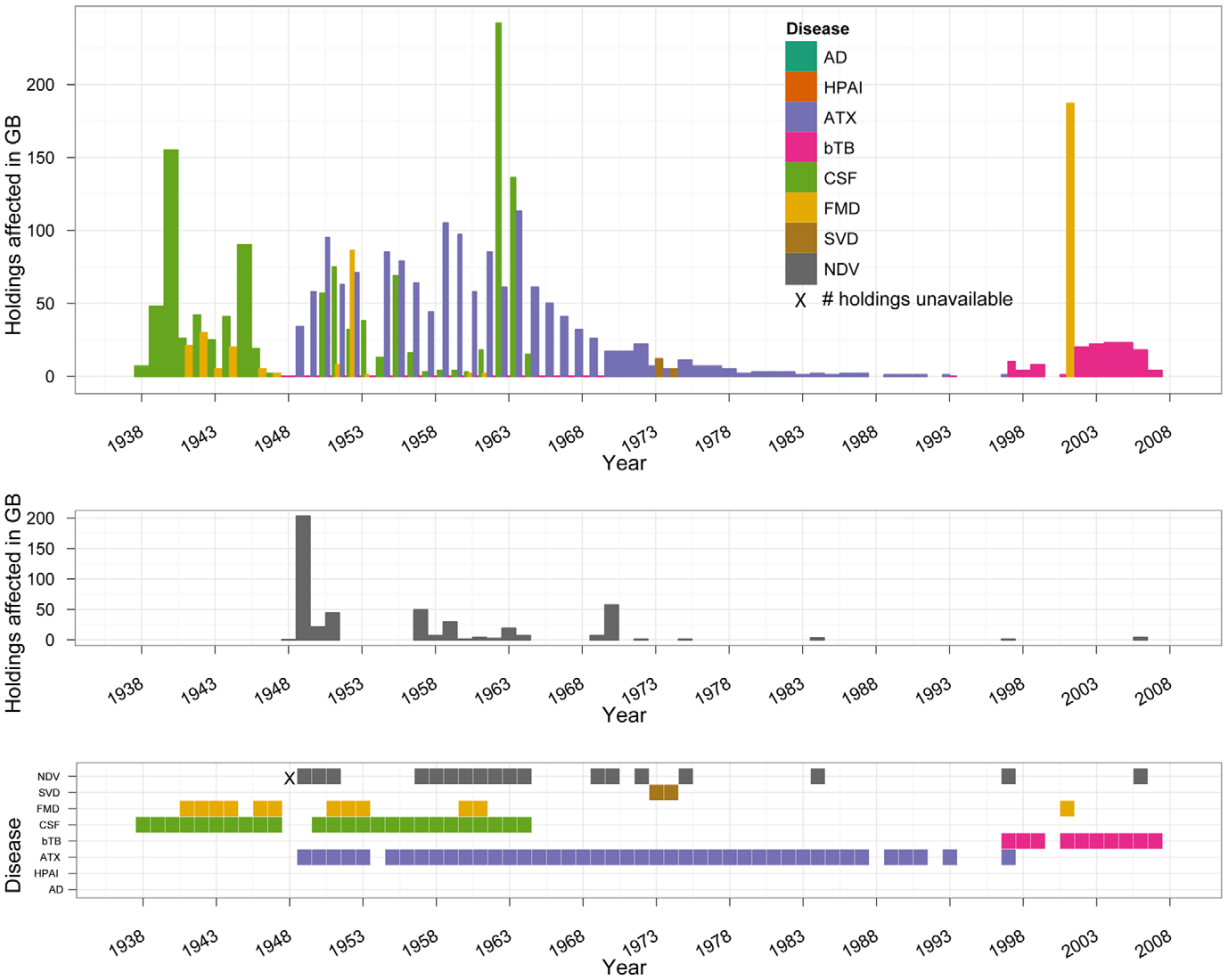
Disease frequency and numbers of holdings affected by reviewed diseases in Scotland 1938–2007. The numbers of new breakdowns with bTB were consistently available only for the years 1997–2007. AD - Aujeszky's disease, ATX-anthrax.

The occurrence of and the numbers of holdings affected by diseases reviewed in Scotland from 1938 to 2007 are summarized in Figure 4, with the exception of bTB for which the limited data availability prevented an evaluation of its occurrence in Scotland prior to 1997.

The frequency of occurrence of diseases reviewed in Scotland dropped during and after the 1970s (Figure 4), despite the rising numbers of sheep and poultry farmed (Figure 1, right panels). These processes were similar to those observed in the rest of GB. However, in the later part of the review period the frequency of diseases in Scotland differed from the rest of GB, with the individual patterns varying by disease (Table 5; Figure 4 versus Figure 2).

**Table 5:** Numbers of years between 1938 and 2007 each reviewed disease was reported in farm-animals in Great Britain as a whole and in Scotland specifically.

disease	years in GB out of 70 years reviewed	years in Scotland out of 70 years reviewed
HPAI	5	0
anthrax	62	43
Aujeszky's disease	11	0
FMD	32	12
CSF	33	25
NDV	36	20
SVD	10	2
bTB	60 of 60 between 1948 and 2007	data inadequate, at least 33 between 1948 and 2007

Foot and mouth disease was reported in GB in 32 of the 70 years between 1938 and 2007; the affected areas included Scotland in only 12 of these 32 years (Table 5). Between 1938 and 1962 FMD was practically still endemic in GB, and occurred south of the Scottish border each year. In 11 of these 25 years the disease reached Scotland; the number of affected Scottish holdings exceeded 30 only in 1952 (Figure 4, top panel). Of the 7 years post-1963 in which FMD was reported in GB, the disease reached Scotland once, in 2001 (Figure 4, bottom panel; versus Figure 2, bottom panel). The maximum number of holdings affected by FMD in Scotland over the 70 years reviewed was 187, recorded in 2001 (Figure 4, top panel).

Classical swine fever was reported in GB in 33 of the 70 years reviewed; the affected areas included Scotland in 25 of these 33 years (Table 5). Maximum incidence of CSF in GB was observed in 1940, with 1.09% of agricultural holdings affected (Table 1). The maximum incidence in Scotland was 0.40%, and occurred much later – in 1962 (Table 6). However, none of the post-1966 outbreaks of CSF in GB involved Scotland (Figure 4, bottom panel; versus Figure 2, bottom panel). SVD was reported in Scotland in 2 of the 10 years that it affected GB. Aujeszky's disease occurred in GB for 11 years but was not reported in Scotland. In summary, of the 19 years that swine exotic diseases occurred in GB post-1966, the affected areas included Scotland in 2 years - 1973 and 1974 (Figure 4, bottom panel; versus Figure 2, bottom panel).

**Table 6:** Maximal annual incidence of reviewed diseases on Scottish agricultural holdings 1938–2007^a^.

disease	year of maximal incidence	annual incidence, %
CSF	1962	0.40
FMD	2001	0.38
NDV	1949	0.27
anthrax	1964	0.19
SVD	1973	0.025

a The locations reported for the five outbreaks of HPAI in Great Britain 1938–2007 were outside Scotland. Aujeszky's disease did not occur in Scotland. Bovine tuberculosis is not included because the numbers of new breakdowns in Scotland were consistently available only for 1997–2007.

Based on the available evidence, of the diseases reviewed, CSF affected the largest number of holdings in Scotland in a single year between 1938 and 2007, with 242 outbreaks in 1962 (Figure 4, top panel). It was followed by NDV with 203 outbreaks in 1949, and FMD with 187 outbreaks in 2001 (Figure 4, top panel). (The number of holdings affected by NDV in Scotland in 1948 was not available, there being 267 in total in GB). Notably, between 1950 and 1967 anthrax was reported on more than 40 holdings in Scotland in all but one year (1954), with the maximum of 113 holdings affected in 1964 (Figure 4, top panel).

Over the 70 years reviewed, the maximal annual incidence of any given exotic disease on agricultural holdings in Scotland was less than half the maximum incidence reported for GB as a single epidemiological unit (Table 6 versus Table 1). In particular, CSF affected a maximum of 0.40% of Scottish holdings in 1962, followed by FMD on 0.38% of holdings in 2001, and NDV on 0.27% of holdings in 1949 (Table 6). The maximum annual incidence of CSF on the scale of GB was 1.09%, of FMD 0.77%, and of NDV 1.50% of agricultural holdings (Table 1). Notably, following these viral diseases, the next highest annual incidence in Scotland was observed for anthrax, with 0.19% of holdings affected in 1964 (Table 6). Considering disease significance by decade, the disease with maximal cumulative annual incidence in Scotland in a given decade was different from that for GB as a whole (Table 7 versus Table 3).

**Table 7:** Reviewed disease with maximal annual incidence on Scottish agricultural holdings in each decade 1938–2007^a^.

period	disease with maximal annual incidence	year of maximal annual incidence	annual incidence, %
1938–1949	NDV	1949	0.27
1950s	anthrax	1959	0.17
1960s	CSF	1962	0.40
1970s	NDV	1970	0.11
1980s	anthrax	1982	0.006
	NDV	1984	0.006
1990s	bTB	1997	0.02
2000–2007	FMD	2001	0.38

a The locations reported for the five outbreaks of HPAI in Great Britain 1938–2007 were outside Scotland. Aujeszky's disease did not occur in Scotland. Bovine tuberculosis is not included because the numbers of new breakdowns in Scotland were consistently available only for 1997–2007.

The only reviewed disease reported in Scotland from 2002 to 2007 was bTB. Between 1997 and 2007, the annual incidence of bTB on agricultural holdings in Scotland ranged from 0.008% to 0.045%, being much lower than the overall rates for GB of 0.20%–0.74% (Figure 3, left panels).

The livestock or avian species reported as being affected in Scotland by viral diseases reviewed mostly mirrored those elsewhere in GB. The percentages of susceptible farm-animals culled in control of the diseases in Scotland were not calculated because the data on the regional culls were unavailable. The location of affected holdings within Scotland, the month of the index case, and the availability of the data pertaining to both of these are summarized in *Table S7*.

Sale of infected animals via livestock markets was most often mentioned as the method through which a livestock disease occurring elsewhere in GB was introduced into Scotland during the review period; the movement of poultry, including incidents of illegal movements, was mentioned for NDV. Recently, following the introduction of bluetongue virus into GB in 2008, a risk assessment of the modes of introduction of a vector-borne livestock disease into Scotland was carried out [19]. It showed that import of infected animals outside vector-free periods (which can occur despite the control measures in place) and arrival of infected vectors from a disease focus in the north of England both pose a relatively high risk. The disease focus can form either inadvertently ahead of the main disease front, or via gradual disease spread north-wise within GB.

### Percentages of holdings farming individual livestock species or poultry affected by reviewed diseases in Great Britain and in Scotland 1938–2007

**Figure 5:**
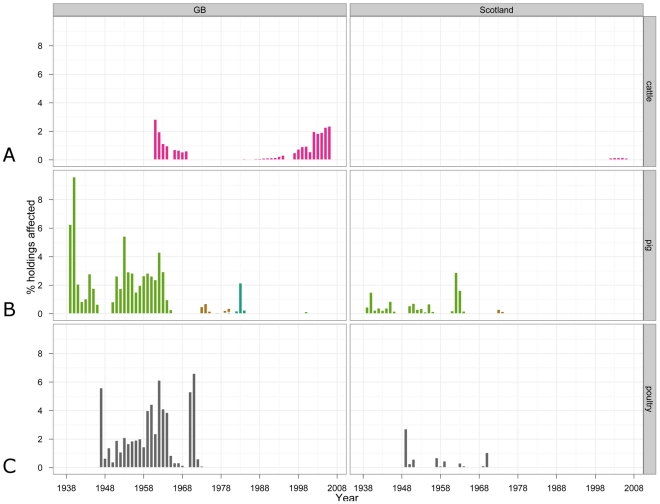
Industry-specific annual incidence in Great Britain (left panels) and in Scotland (right panels) of: (A) bovine tuberculosis on cattle holdings 1948–2007; (B) Aujeszky's disease, classical swine fever and swine vesicular disease on pig holdings 1938–2007; and (C) Newcastle disease on poultry holdings 1947–2007. The numbers of herd breakdowns with bTB in GB were included in the reports of British governmental veterinary services since 1948, and the numbers of NDV outbreaks since 1947. AD - Aujeszky's disease, ATX-anthrax.

The percentages of pig holdings in GB affected by swine diseases reviewed and of poultry holdings affected by NDV throughout the 70 years are given in Figure 5, left panels; the same statistics for Scotland are given in Figure 5, right panels. The percentages of cattle holdings in GB affected by bTB are given in the top left panel of Figure 5; but this plot must be read with caution, considering the amount of missing data on the numbers of affected holdings as detailed in the bottom panel of Figure 2. Overall, the results in Figure 5 should be interpreted only as a guide, considering limitations of the denominator data (see *Text S2. Data sources, availability and limitations* for details).

## Discussion

The aim of this work was to systematically review the occurrence of the diseases of the former OIE List A and of three other major infectious diseases of farm animals in GB as a single epidemiological unit, and in Scotland specifically, over the 70 years 1938–2007. The results of the review cover eight individual diseases reported in GB in those 70 years. We believe that the figures and tables presenting the results allow the reader to evaluate the trends or extract the statistics for a disease of interest. We therefore do not discuss the trends observed for individual diseases. Neither does the format of a single paper allow for an in-depth discussion of the complex epidemiology of each disease. Instead, in this discussion we concentrate on the general patterns in the frequency and incidence of exotic diseases in Britain and in Scotland specifically, and on the factors that affected disease occurrence. We also discuss how the limitations of the available data affect our results.

The occurrence of the reviewed diseases in GB as a whole, and in Scotland specifically, from 1938 to 2007 was evaluated with several indexes. First, the annual incidence of the diseases on agricultural holdings was estimated. This denominator allowed a comparative evaluation of the incidence of diseases affecting different species of farm-animals, “per holding risks”, and the importance of individual diseases over the years. These denominator data were also consistently available throughout the review period. In this denominator, however, the holdings of different production types and sizes were counted equally. The structure of the British farm-animal industry changed over the 70 years studied. For example, in Wales between 1974 and 1995, the number of larger holdings (those with total land ≥100 hectares) increased, while the number of small holdings (10–30 hectares) decreased [20]. In GB as a whole, the amalgamation of agricultural production onto larger holdings occurred mainly over a period from the 1950s through to the 1980s; in Scotland, this occurred between the late 1950s and the 1960s; as evident in Figure 1. The economic and social consequences of the diseases in individual years depended upon which types of holdings were affected, and whether the breeding stock or other parts of livestock populations were affected. Which segments are affected also determines how the diseases impact on the social stability of countryside communities whose livelihoods rely on animal farming. With the available data, it was not possible to differentiate the impact of reviewed diseases on the various segments of British livestock industries. Consistent recording of such information would improve the analysis of disease outbreaks and control measures.

The estimation of the annual disease incidence was complemented by the estimation of the fraction of susceptible farm-animals culled in control of the disease in GB each year reviewed. This provided additional information on the scale of the disease episodes; their relative impacts on livestock sectors; and effects on animal welfare, *i.e.* the per capita likelihood for susceptible livestock farmed to succumb to or to survive a disease episode in different years. However, again, the broader economic and social consequences of the diseases depended on the importance of the sectors affected. For example, in the 1950s–1980s outbreaks of viral swine diseases led to the largest fractional culls of susceptible livestock in GB in individual years (Table 4). However, the pig sector was relatively small compared to the headage of sheep or cattle (Figure 1; left panels). Therefore, the net consequences of swine diseases for society as a whole may have been limited. In contrast, the largest fractional cull of susceptible livestock between 2002 and 2007 was due to bTB (Figure 3, right panels), at which time the cattle sector was the second largest livestock industry in Britain (Figure 1; left panels). Consequently, the net effect of this single disease was exceptional [21].

The zoonotic potential of a disease is another factor determining its importance for society. This potential varies widely between the diseases reviewed. The viral diseases devastating the British swine industry in the 1950s–1980s had no to little zoonotic potential. In contrast, bTB, observed to be the disease with the highest annual incidence on GB holdings in the absence of the former OIE List A diseases, is zoonotic, and historically was one of the major drivers behind the introduction of pasteurization of milk for human consumption.

Most of the diseases considered in this review are “exotic” for Britain at present; their introduction can have large consequences for the economy and politics of farm industries. However, it must be noted that a long-term impact of endemic diseases may exceed that of a rare occurrence of exotics in terms of animal loss, animal welfare, and overall economic cost. It would be of great interest to compare the impacts of endemics and exotics over years. Unfortunately, the authors are unaware of a consistent source of historical information on the incidence and livestock culls for endemic diseases in Britain.

The ultimate drop in the frequency of FMD, CSF and exotic NDV in GB (Figure 2, bottom panel) was achieved against the background of the amalgamation of agricultural production on larger holdings and the dynamically rising numbers of livestock and poultry farmed (Figures 1, left panels).

The specific measures, including vaccination campaigns, introduced to control CSF, FMD and NDV in GB before 1965 are detailed in the book “Animal Health: A centenary 1865–1965. A century of endeavour to control diseases of animals” [11]. The timeline of major general disease control measures introduced in GB between 1938 and 2007, which in our opinion have impacted on the occurrence of exotics, is given in Table 8. We conjecture that at least three other sets of factors have significantly impacted on the chances for disease occurrence: those resulting from re-structuring of the farm industries, the successes of biotechnology, and the theory of infectious disease control. A combination of forces, beyond the scope of this discussion, was driving the amalgamation of agricultural production. Nonetheless, the resulting larger holdings were able to afford better biosecurity. This was accompanied by the emergence of pyramidal production structures within the swine and poultry industries, with the separated breeder sector adopting high biosecurity standards. At the same time, biotechnology has seen a number of successes, resulting in the development and roll-out of mass-application serological diagnostics tools and vaccines to combat animal infections. The serological diagnostics enabled the identification of those infected and prompt targeting of control. Epidemiological theory has evolved and advanced, *e.g.*, the notion of the sufficient fractional vaccination coverage necessary for regional control of a viral disease of farm-animals became widely accepted by the early 1960s [11]. All of these factors, together with the general (Table 8) and specific disease control measures enforced or promoted by the governments, were major contributors to the first-time eradication of CSF and FMD from GB in the late 1960s, and the prevention of exotic NDV in commercial poultry from the mid-1970s onwards.

**Table 8:** Timeline of introduction of major general disease control measures with potential to affect the occurrence of exotics in Great Britain between 1938 and 2007^a^.

year	measure introduced
1968	ban on import of in-bone meat from South America
1975	order on movement and sale of pigs
1988	ban on feeding ruminant protein to ruminants
1996	passports for cattle
1997	order requiring disinfection of vehicles for animal transport
1998	individual cattle movement tracing (reporting and recording) system
1999	ban on feeding animal by-products to farm-animals
2000	updated order on disinfection of vehicles for animal transport
2001	ban on feeding swill to livestock
2002	standstill period for livestock on the farm following an on-movement of livestock
2002	tracing systems for movements of batches of sheep, pigs, goats and farmed deer

a For the measures introduced before 1938 that may have affected the disease occurrence during the review period see Animal Health: A centenary 1865–1965. A century of endeavour to control diseases of animals. Her Majesty's Stationery Office, London, 1965.

From the available data, it is evident that the frequency of occurrence and the maximal annual incidence of exotic diseases in Scotland between 1938 and 2007 were lower compared to the rest of GB. (The livestock or avian species reported to be affected by viral diseases reviewed in Scotland mostly mirrored those elsewhere in GB.) The factors underlying these apparent differences are likely to be multi-fold. At the very least, they include the differences in risks of disease introduction, and in probabilities of detecting and reporting an affected holding. The risks of disease introduction vary due to geography and farm-animal trading practices. For example, imports of livestock from continental Europe to England may be more frequent than to Scotland, due to both closer proximities and trading traditions. Livestock imports from Ireland to Scotland, on the other hand, may be more frequent than those to England, for the same reasons, *e.g.* the import of Irish cattle to Scotland [22]. Within Great Britain, Scotland's historical position as a net exporter of sheep to England lowers the chances for a disease moving northwards [23]–[25]; although, remembering the FMD outbreak in the UK in 2001, these chances are not negligible. Also, during 2003–2007 Scotland has been a net importer of beef and dairy cattle from the rest of Britain [25]. The probabilities of detecting and reporting a holding affected by a given disease in Scotland may differ from the rest of GB due to varying livestock demographics. For example, throughout the 70 years reviewed, sheep constituted a larger fraction of total livestock in Scotland compared to that elsewhere in GB (Figure 1, bottom panels). The course of FMD in sheep can result in only limited pathology, and an outbreak of FMD on a sheep-only holding may either take longer to detect [26] or resolve without detection. However, although the demographics could have affected the scale of incidence reported for reviewed diseases, it is unlikely that the frequency of the disease episodes in Scotland during the review period was under-reported.

In terms of the month of the index case, the available evidence suggests that there might be a higher risk of FMD introduction into Scotland between March and May (*Table S7*). At this time of year during 2003–2007 there was a secondary annual peak in the numbers of sheep moved between the farms on mainland Scotland, and moved onto the island parishes of Argyll and Bute (with the primary peak occurring in September) [25]. In addition, on average larger batches of sheep were moved between the farms on the mainland compared to the rest of the year (unpublished data). The description of sheep movements in Scotland in the 1960s [23], report a spring peak in sales of sheep for breeding. Also, at this time of the year during 2003–2007, relatively high numbers of beef cattle were moved cross-border from England into Scotland [25]. These patterns may have remained stable throughout the years reviewed, and therefore the period of a higher risk of FMD introduction may have coincided with the period of a higher risk of subsequent spread of disease within Scotland. However, intensive livestock movement simultaneously poses a risk of infection transmission and an increased chance for disease detection (as the animals are likely to be examined during the market sale and on the holdings of departure and destination) [26]. Therefore, a higher frequency of the index case of FMD in Scotland in the spring may be also reflecting the more frequent detection circumstances.

The interpretation of the results discussed here is subject to limitations of the available data on disease occurrence and denominator livestock populations, which are detailed in *Text S2. Data sources, availability and limitations*. We would also like to comment that the denominators (numbers of agricultural holdings and of animals on them) were extracted from the results of British agricultural censuses, which are based on County-Parish-Holding (CPH) identifiers. Each CPH is counted as a holding, and the numbers of animals farmed are reported for each CPH. Although this identifier had been used throughout the review period [27], over time the CPH system as the source of data on livestock location come to suffer from a number of limitations, and alternative systems are being considered [28]. First, mandatory reporting of movements of individual cattle to a central database was introduced in GB in 1999; mandatory reporting of movements of batches of sheep, pigs, goats and farmed deer followed in 2002 (Table 8). These regulations included an exemption permitting to not report livestock movements if the source and destination holdings belonged to the same business [27]. Industry's adaptation led to documenting “distance-linked” CPH, that is – farms located far apart (sometimes hundreds kilometres apart) but owned by the same business are documented under a single CPH [27], allowing to not report livestock movements between the farms. This may have biased our calculations of the numbers of agricultural holdings in GB and in Scotland alone, and so the calculations of annual disease incidence in GB and in Scotland, and of numbers of livestock farmed in Scotland between 2002 and 2007. For example, there may have been a higher number of farms in Scotland in a given year compared to Figure 1, right panels, since >1 farm could have been reported under a single CPH; therefore the disease incidence for that year may be an overestimate. Or the animals counted to be in Scotland based on the holding's CPH, may have been located somewhere in England, or *vice versa*, resulting in numbers in Figure 1, right panels, being either over- or under-estimates, respectively. Other shortcomings of the CPH system which may have similarly biased our results are failure to report a holding [29], and the CPH designating the farm office or farmer's house with the animals being kept on other lands if this involved the border between England and Scotland [29], [30]. Farm subsidies (up until the 2000s based on the headage of livestock on the farm) was a driver affecting how the farm lands were reported [30] prior to the livestock movement regulations, but with less dramatic effects. Currently proposed alternatives for a livestock holding identifier in GB include defining Livestock Movement Units [27], or providing a unique identifier for lands within no more than a 10 mile radius [28]; the latter is close to how original CPH identifiers were allocated in the 1940s [27]. The completeness and reliability of the data on livestock location determines how well an outbreak of infectious disease is traced and controlled [31], as well as the quality of its historical analysis.

Another limitation is that when evaluating the fractions of susceptible livestock culled in disease control, only cattle, sheep and pigs farmed were counted. Less common farm stock, such as goats or deer, were not counted due to the lack of historical data. This approximation was probably acceptable for the 70 years reviewed, given the composition of livestock species farmed. For example, the number of cattle farmed in England and Wales in a given year 1991–1999 was 7.7–8.2 million; the number of goats was 64,700–80,000; and the number of farmed deer was 25,400–39,100. In the same period, the number of cattle farmed in Scotland was around 2.1 million; the number of goats decreased from about 20,000 in 1991–1992 to 8,900 in 1999; and the number of farmed deer varied from 6,600 to 13,000 depending on the year.

The demographics of farm-animal populations susceptible to individual exotic diseases may change in the future. The diversification of production on individual agricultural holdings, the changing physical and political climates, and the growing demand for food will all determine whether the growth in the numbers of livestock farmed in Britain returns to the rates seen in the 1980s and the 1990s. Change in costumer preference, *i.e.* growing demand for organic products, will likely lead to further re-structuring of the industry, and perhaps to opposite trends compared with the intensification and amalgamation of production experienced during the years reviewed. (For a projection of trends in British livestock demographics see Bessell *et al.* (2006) [32]). The trends in livestock demographics need to be analyzed for their impact on the risks of exotic disease introduction and subsequent spread. The differences in policies between Scotland and the rest of GB in the future may lead to even bigger differences in the risks of exotics, and the significance of individual diseases than those observed between 1938 and 2007.

To conclude, the measures in place today are primarily oriented on prevention of introduction and fast detection of exotic animal disease in GB. However a lot can be learned from the great successes in control of contagious livestock and poultry diseases in the 1960s and the 1970s, both in terms of the methods for keeping exotics out of the British isles, and for dealing with the outbreaks which will inevitably occur. As a Russian saying goes, “everything new is a well-forgotten old”. An in-depth analysis of the relative contribution of individual factors (governmental policies, improved diagnostics and vaccines and epidemiological theory of their application, pyramidal structures of the pig and poultry industries, and increased biosecurity standards) to those successes is worthy of consideration.

From the results of the present review, there is historical evidence that separated disease statistics for Scotland are informative. Between 1938 and 2007, exotic diseases of farm-animals were reported in Scotland less often than in the rest of GB, and with a lower annual incidence during the disease episodes. From a historical point of view, the regionalization of control measures (*e.g.* vaccination coverage or zoning) between Scotland and the rest of Britain during outbreaks of exotic diseases of farm-animals appears to be well-grounded.

## Supporting Information

Table S1 **Denominator populations in Great Britain: numbers of sheep, cattle, pigs, total main livestock, total poultry, and number of agricultural holdings each year 1938–2007.** Total main livestock is defined as the sum of sheep, cattle and pigs farmed.
(DOC)

Table S2 **Denominator populations in Scotland: numbers of sheep, cattle, pigs, total main livestock, total poultry, and number of agricultural holdings each year 1938–2007.** Total main livestock is defined as the sum of sheep, cattle and pigs farmed.
(DOC)

Table S3 **Reviewed disease with maximal incidence on agricultural holdings of Great Britain each year 1938–2007.**
(DOC)

Table S4 **Reviewed disease due to which maximal percentage of susceptible farm-animals was culled in Great Britain each year 1938–2007.**
(DOC)

Table S5 **Livestock disease reviewed due to which the maximal percentage of susceptible animals farmed in Great Britain was culled each year 1938–2007.**
(DOC)

Table S6 **Total percentage of cattle, sheep and pigs farmed in Great Britain culled in control of the reviewed diseases each year 1938–2007.**
(DOC)

Table S7 **The month of index case and the locality of affected holdings in Scotland.**
(DOC)

Text S1 **Conduct of the systematic review.**
(DOC)

Text S2 **Data sources, availability and limitations.**
(DOC)
