# Correction: A Review of Exotic Animal Disease in Great Britain and in Scotland Specifically between 1938 and 2007

**DOI:** 10.1371/annotation/98e38a89-ff2c-4242-b8bd-2920676ef68b

**Published:** 2011-08-08

**Authors:** Onneile O. Peiso, Barend M. de C. Bronsvoort, Ian G. Handel, Victoriya V. Volkova

Reference is missing a book title. The complete Reference 10 is: 10. Gannushkin MS (1961) (Book in Russian). Общая эпизоотология (General epizootology) Moscow: State Publishing House of Agricultural Literature

