# Correction: A Review of Exotic Animal Disease in Great Britain and in Scotland Specifically between 1938 and 2007

**DOI:** 10.1371/annotation/5243392a-04fb-4ff7-9cee-e4adcc25380e

**Published:** 2011-08-08

**Authors:** Onneile O. Peiso, Barend M. de C. Bronsvoort, Ian G. Handel, Victoriya V. Volkova

The color legend is missing from the bottom part of Figure 1. The correct Figure 1 can be viewed here: 

**Figure:**
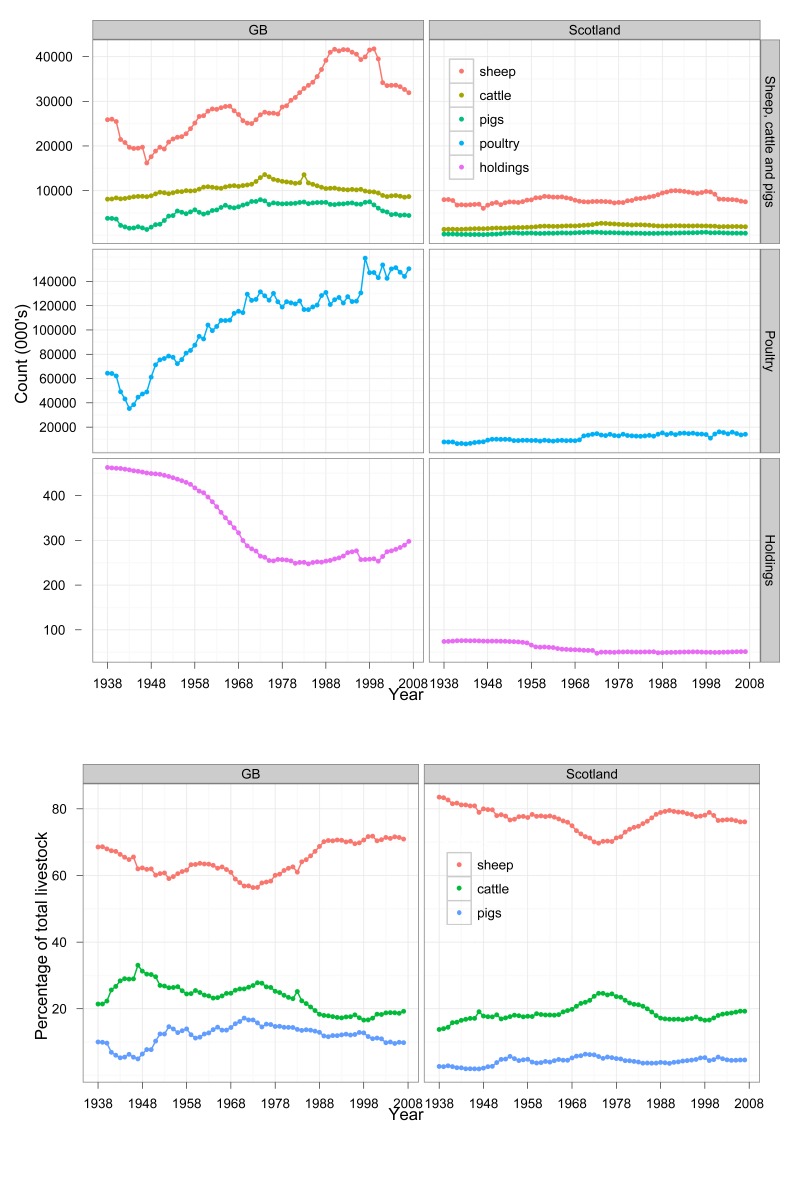


f 

