# Supplementary material for: A Review of Exotic Animal Disease in Great Britain and in Scotland Specifically between 1938 and 2007
Source: PLoS One. 2011 Jul 27;6(7):e22066. doi: 10.1371/journal.pone.0022066 (PMC3144883; doi:10.1371/journal.pone.0022066)
Supplement: Table S1 — Denominator populations in Great Britain: numbers of sheep, cattle, pigs, total main livestock, total poultry, and number of agricultural holdings each year 1938–2007. Total main livestock is defined as the sum of sheep, cattle and pigs farmed. (DOC) [file pone.0022066.s001.doc]

**Table S1.** Denominator populations in Great Britain: numbers of sheep, cattle, pigs, total main livestock, total poultry, and number of agricultural holdings each year 1938-2007. Total main livestock is defined as the sum of sheep, cattle and pigs farmed.

| year | sheep | cattle | pigs | total main livestock | poultry | agricultural holdings |
| --- | --- | --- | --- | --- | --- | --- |
| 1938 | 25,874,454 | 8,085,922 | 3,787,477 | 37,747,853 | 64,385,616 | 462,735 |
| 1939 | 25,992,759 | 8,118,788 | 3,767,301 | 37,878,848 | 64,136,844 | 461,771 |
| 1940 | 25,465,062 | 8,361,123 | 3,631,074 | 37,457,259 | 62,121,087 | 460,958 |
| 1941 | 21,445,117 | 8,153,176 | 2,207,227 | 31,805,520 | 49,125,980 | 460,552 |
| 1942 | 20,763,940 | 8,248,055 | 1,871,573 | 30,883,568 | 43,212,182 | 458,961 |
| 1943 | 19,699,614 | 8,427,727 | 1,571,461 | 29,698,802 | 35,299,049 | 457,492 |
| 1944 | 19,435,396 | 8,615,580 | 1,630,515 | 29,681,491 | 38,481,116 | 455,408 |
| 1945 | 19,495,536 | 8,697,169 | 1,902,973 | 30,095,678 | 44,665,142 | 454,300 |
| 1946 | 19,718,138 | 8,715,527 | 1,643,869 | 30,077,534 | 47,275,973 | 452,512 |
| 1947 | 16,186,297 | 8,633,424 | 1,294,186 | 26,113,907 | 48,977,093 | 450,508 |
| 1948 | 17,589,068 | 8,839,699 | 1,815,742 | 28,244,509 | 61,138,123 | 449,213 |
| 1949 | 18,847,312 | 9,263,464 | 2,364,147 | 30,474,923 | 71,256,654 | 448,465 |
| 1950 | 19,713,558 | 9,630,276 | 2,462,589 | 31,806,423 | 75,385,009 | 447,566 |
| 1951 | 19,311,429 | 9,512,234 | 3,306,104 | 32,129,767 | 76,505,580 | 445,245 |
| 1952 | 20,859,819 | 9,302,617 | 4,286,628 | 34,449,064 | 78,518,740 | 442,763 |
| 1953 | 21,560,067 | 9,517,543 | 4,406,418 | 35,484,028 | 77,511,426 | 439,861 |
| 1954 | 21,942,673 | 9,776,796 | 5,430,862 | 37,150,331 | 72,258,355 | 436,630 |
| 1955 | 22,078,493 | 9,764,170 | 5,157,109 | 36,999,772 | 75,585,082 | 433,167 |
| 1956 | 22,721,266 | 9,989,372 | 4,820,536 | 37,531,174 | 80,927,992 | 429,484 |
| 1957 | 23,867,705 | 9,908,770 | 5,232,473 | 39,008,948 | 83,131,216 | 425,057 |
| 1958 | 25,125,449 | 9,976,375 | 5,695,044 | 40,796,868 | 87,474,148 | 417,178 |
| 1959 | 26,600,557 | 10,327,575 | 5,135,436 | 42,063,568 | 94,718,374 | 409,988 |
| 1960 | 26,772,048 | 10,772,102 | 4,739,299 | 42,283,449 | 92,611,976 | 406,244 |
| 1961 | 27,783,858 | 10,860,667 | 5,009,407 | 43,653,932 | 104,074,897 | 396,794 |
| 1962 | 28,288,930 | 10,749,223 | 5,539,984 | 44,578,137 | 99,435,717 | 386,206 |
| 1963 | 28,203,798 | 10,605,232 | 5,669,662 | 44,478,692 | 102,878,846 | 375,111 |
| 1964 | 28,563,026 | 10,514,939 | 6,225,546 | 45,303,511 | 107,820,052 | 362,456 |
| 1965 | 28,836,801 | 10,826,334 | 6,711,365 | 46,374,500 | 107,746,494 | 350,426 |
| 1966 | 28,902,750 | 11,017,257 | 6,276,416 | 46,196,423 | 108,075,975 | 339,379 |
| 1967 | 27,873,790 | 11,106,209 | 6,131,385 | 45,111,384 | 113,680,029 | 328,142 |
| 1968 | 27,041,786 | 10,943,617 | 6,374,619 | 44,360,022 | 115,400,088 | 316,806 |
| 1969 | 25,669,008 | 11,130,835 | 6,750,194 | 43,550,037 | 114,277,644 | 299,551 |
| 1970 | 25,113,639 | 11,261,423 | 7,019,673 | 43,394,735 | 129,463,740 | 287,664 |
| 1971 | 25,005,585 | 11,420,283 | 7,566,799 | 43,992,667 | 124,352,059 | 281,010 |
| 1972 | 25,872,795 | 12,039,675 | 7,571,851 | 45,484,321 | 125,174,702 | 276,073 |
| 1973 | 26,979,135 | 12,909,547 | 7,964,651 | 47,853,333 | 131,385,838 | 264,684 |
| 1974 | 27,561,880 | 13,583,227 | 7,704,800 | 48,849,907 | 128,109,203 | 262,089 |
| 1975 | 27,335,789 | 13,091,114 | 6,886,207 | 47,313,110 | 124,515,120 | 254,900 |
| 1976 | 27,338,858 | 12,521,761 | 7,249,234 | 47,109,853 | 130,113,244 | 254,002 |
| 1977 | 27,153,368 | 12,288,723 | 7,111,201 | 46,553,292 | 123,215,849 | 257,342 |
| 1978 | 28,711,939 | 12,076,336 | 7,024,253 | 47,812,528 | 118,873,867 | 256,823 |
| 1979 | 28,997,131 | 11,939,674 | 7,077,188 | 48,013,993 | 123,252,470 | 255,958 |
| 1980 | 30,220,422 | 11,814,742 | 7,100,152 | 49,135,316 | 122,311,217 | 254,040 |
| 1981 | 30,854,126 | 11,629,623 | 7,156,630 | 49,640,379 | 121,418,987 | 248,640 |
| 1982 | 31,941,252 | 11,753,903 | 7,339,743 | 51,034,898 | 123,878,167 | 250,644 |
| 1983 | 32,864,938 | 13,561,457 | 7,440,066 | 53,866,461 | 116,827,334 | 250,821 |
| 1984 | 33,571,591 | 11,718,814 | 7,075,363 | 52,365,768 | 116,735,313 | 247,424 |
| 1985 | 34,256,367 | 11,408,845 | 7,249,412 | 52,914,624 | 118,960,493 | 250,471 |
| 1986 | 35,534,327 | 11,075,104 | 7,321,084 | 53,930,515 | 120,507,517 | 251,978 |
| 1987 | 37,117,292 | 10,752,624 | 7,337,476 | 55,207,392 | 128,339,610 | 251,203 |
| 1988 | 39,153,063 | 10,456,755 | 7,363,727 | 56,973,545 | 130,863,963 | 253,608 |
| 1989 | 40,978,791 | 10,520,425 | 6,921,166 | 58,420,382 | 120,962,810 | 255,147 |
| 1990 | 41,644,864 | 10,566,537 | 6,860,993 | 59,072,394 | 124,878,060 | 258,327 |
| 1991 | 41,276,577 | 10,347,003 | 7,010,306 | 58,633,886 | 126,744,594 | 260,742 |
| 1992 | 41,578,937 | 10,242,825 | 7,023,348 | 58,845,110 | 122,207,971 | 264,897 |
| 1993 | 41,525,121 | 10,165,837 | 7,162,320 | 58,853,278 | 127,404,185 | 272,417 |
| 1994 | 40,992,171 | 10,266,693 | 7,238,523 | 58,497,387 | 123,375,869 | 274,330 |
| 1995 | 40,550,348 | 10,157,446 | 6,990,045 | 57,697,839 | 123,731,925 | 276,518 |
| 1996 | 39,332,591 | 10,280,287 | 6,951,841 | 56,564,719 | 130,504,329 | 256,958 |
| 1997 | 39,942,930 | 9,901,551 | 7,375,393 | 57,219,874 | 159,111,921 | 257,227 |
| 1998 | 41,484,462 | 9,751,478 | 7,493,062 | 58,729,002 | 147,144,625 | 257,803 |
| 1999 | 41,747,364 | 9,704,824 | 6,793,623 | 58,245,811 | 147,268,510 | 258,806 |
| 2000 | 39,488,538 | 9,449,820 | 6,059,585 | 54,997,943 | 143,047,348 | 253,367 |
| 2001 | 34,176,193 | 8,921,137 | 5,442,486 | 48,539,816 | 153,541,051 | 263,873 |
| 2002 | 33,510,107 | 8,660,726 | 5,200,323 | 47,371,156 | 142,509,697 | 274,488 |
| 2003 | 33,570,443 | 8,822,894 | 4,612,093 | 47,005,429 | 150,246,213 | 276,508 |
| 2004 | 33,592,299 | 8,896,195 | 4,736,703 | 47,225,198 | 151,290,834 | 279,914 |
| 2005 | 33,270,895 | 8,737,001 | 4,456,855 | 46,464,751 | 147,527,974 | 283,970 |
| 2006 | 32,652,034 | 8,528,398 | 4,546,324 | 45,726,756 | 144,083,251 | 289,141 |
| 2007 | 31,921,829 | 8,660,524 | 4,423,925 | 45,006,278 | 150,381,975 | 297,703 |
