# Supplementary material for: A Review of Exotic Animal Disease in Great Britain and in Scotland Specifically between 1938 and 2007
Source: PLoS One. 2011 Jul 27;6(7):e22066. doi: 10.1371/journal.pone.0022066 (PMC3144883; doi:10.1371/journal.pone.0022066)
Supplement: Table S2 — Denominator populations in Scotland: numbers of sheep, cattle, pigs, total main livestock, total poultry, and number of agricultural holdings each year 1938–2007. Total main livestock is defined as the sum of sheep, cattle and pigs farmed. (DOC) [file pone.0022066.s002.doc]

**Table S2**. Denominator populations in Scotland: numbers of sheep, cattle, pigs, total main livestock, total poultry, and number of agricultural holdings each year 1938-2007. Total main livestock is defined as the sum of sheep, cattle and pigs farmed.

| year | sheep | cattle | pigs | total main livestock | poultry | agricultural holdings |
| --- | --- | --- | --- | --- | --- | --- |
| 1938 | 7,969,482 | 1,315,731 | 257,374 | 9,542,587 | 7,778,969 | 74,017 |
| 1939 | 8,007,100 | 1,348,643 | 252,200 | 9,607,943 | 7,710,990 | 74,291 |
| 1940 | 7,782,532 | 1,360,123 | 271,489 | 9,414,144 | 7,741,442 | 74,801 |
| 1941 | 6,759,334 | 1,311,765 | 218,257 | 8,289,356 | 6,416,793 | 75,719 |
| 1942 | 6,831,443 | 1,335,133 | 193,104 | 8,359,680 | 6,530,455 | 75,779 |
| 1943 | 6,766,415 | 1,377,474 | 190,248 | 8,334,137 | 6,177,922 | 75,879 |
| 1944 | 6,833,395 | 1,417,753 | 167,600 | 8,418,748 | 6,607,373 | 75,474 |
| 1945 | 6,899,009 | 1,459,792 | 170,564 | 8,529,365 | 7,313,087 | 75,716 |
| 1946 | 6,953,914 | 1,472,013 | 169,492 | 8,595,419 | 7,708,683 | 75,324 |
| 1947 | 6,024,688 | 1,458,604 | 148,288 | 7,631,580 | 7,916,803 | 74,878 |
| 1948 | 6,730,664 | 1,499,243 | 183,700 | 8,413,607 | 9,284,741 | 74,726 |
| 1949 | 7,102,873 | 1,568,570 | 232,445 | 8,903,888 | 10,006,270 | 74,873 |
| 1950 | 7,337,269 | 1,616,390 | 250,830 | 9,204,489 | 10,073,107 | 74,792 |
| 1951 | 6,858,883 | 1,600,127 | 339,053 | 8,798,063 | 9,920,969 | 74,606 |
| 1952 | 7,273,407 | 1,575,650 | 446,781 | 9,295,838 | 9,976,118 | 74,444 |
| 1953 | 7,465,866 | 1,656,536 | 470,876 | 9,593,278 | 9,885,551 | 73,980 |
| 1954 | 7,429,375 | 1,709,691 | 552,976 | 9,692,042 | 8,919,138 | 73,590 |
| 1955 | 7,335,648 | 1,724,788 | 479,804 | 9,540,240 | 8,929,548 | 73,026 |
| 1956 | 7,524,598 | 1,736,208 | 431,164 | 9,691,970 | 9,162,285 | 72,175 |
| 1957 | 7,861,976 | 1,779,108 | 473,322 | 10,114,406 | 9,157,731 | 70,745 |
| 1958 | 7,929,302 | 1,819,590 | 495,780 | 10,244,672 | 8,989,034 | 65,948 |
| 1959 | 8,383,659 | 1,892,411 | 428,025 | 10,704,095 | 9,043,631 | 61,911 |
| 1960 | 8,407,076 | 2,002,824 | 402,630 | 10,812,530 | 8,521,519 | 61,308 |
| 1961 | 8,709,479 | 2,044,559 | 430,133 | 11,184,171 | 9,200,102 | 61,750 |
| 1962 | 8,639,227 | 2,016,824 | 466,188 | 11,122,239 | 8,821,225 | 60,953 |
| 1963 | 8,539,130 | 1,989,425 | 439,149 | 10,967,704 | 8,478,083 | 60,444 |
| 1964 | 8,531,203 | 1,989,788 | 486,549 | 11,007,540 | 8,974,521 | 58,258 |
| 1965 | 8,586,642 | 2,035,218 | 533,321 | 11,155,181 | 9,116,945 | 56,835 |
| 1966 | 8,376,838 | 2,090,961 | 500,663 | 10,968,462 | 8,825,298 | 56,344 |
| 1967 | 8,211,902 | 2,103,516 | 492,372 | 10,807,790 | 8,980,259 | 55,770 |
| 1968 | 7,849,179 | 2,077,674 | 549,594 | 10,476,447 | 8,797,010 | 55,487 |
| 1969 | 7,616,976 | 2,152,525 | 600,268 | 10,369,769 | 9,481,980 | 55,148 |
| 1970 | 7,493,866 | 2,233,720 | 611,282 | 10,338,868 | 12,782,190 | 54,256 |
| 1971 | 7,453,879 | 2,283,945 | 663,406 | 10,401,230 | 13,342,324 | 54,060 |
| 1972 | 7,551,791 | 2,387,655 | 661,833 | 10,601,279 | 14,108,329 | 53,930 |
| 1973 | 7,572,596 | 2,565,713 | 667,581 | 10,805,890 | 14,617,243 | 47,738 |
| 1974 | 7,571,044 | 2,675,893 | 616,483 | 10,863,420 | 13,491,448 | 50,196 |
| 1975 | 7,535,591 | 2,641,888 | 549,054 | 10,726,533 | 13,048,386 | 50,286 |
| 1976 | 7,478,173 | 2,568,724 | 587,425 | 10,634,322 | 14,101,769 | 50,107 |
| 1977 | 7,236,896 | 2,522,787 | 549,809 | 10,309,492 | 13,068,956 | 49,667 |
| 1978 | 7,352,307 | 2,441,451 | 521,505 | 10,315,263 | 12,783,174 | 50,619 |
| 1979 | 7,319,086 | 2,397,853 | 504,609 | 10,221,548 | 14,151,876 | 50,580 |
| 1980 | 7,719,565 | 2,383,185 | 468,043 | 10,570,793 | 13,118,623 | 50,943 |
| 1981 | 7,785,556 | 2,291,430 | 463,290 | 10,540,276 | 12,833,793 | 50,504 |
| 1982 | 8,179,827 | 2,345,027 | 462,301 | 10,987,155 | 12,624,890 | 50,376 |
| 1983 | 8,243,226 | 2,337,199 | 443,500 | 11,023,925 | 12,471,969 | 50,409 |
| 1984 | 8,364,564 | 2,299,208 | 406,745 | 11,070,517 | 12,773,481 | 50,638 |
| 1985 | 8,577,634 | 2,247,307 | 419,717 | 11,244,658 | 13,223,028 | 50,821 |
| 1986 | 8,734,110 | 2,151,084 | 414,998 | 11,300,192 | 12,561,107 | 50,993 |
| 1987 | 9,077,094 | 2,079,776 | 428,169 | 11,585,039 | 14,144,129 | 48,730 |
| 1988 | 9,468,733 | 2,062,805 | 468,702 | 12,000,240 | 15,263,475 | 49,073 |
| 1989 | 9,683,624 | 2,074,517 | 463,768 | 12,221,909 | 13,851,083 | 49,546 |
| 1990 | 9,933,721 | 2,106,237 | 451,757 | 12,491,715 | 14,869,195 | 49,689 |
| 1991 | 9,987,040 | 2,122,090 | 495,423 | 12,604,553 | 13,797,410 | 49,860 |
| 1992 | 9,925,650 | 2,122,550 | 510,760 | 12,558,960 | 14,879,760 | 50,248 |
| 1993 | 9,821,000 | 2,076,320 | 536,680 | 12,434,000 | 15,062,380 | 50,489 |
| 1994 | 9,664,880 | 2,091,920 | 547,530 | 12,304,330 | 14,647,480 | 50,635 |
| 1995 | 9,509,360 | 2,073,750 | 558,150 | 12,141,260 | 15,027,830 | 50,843 |
| 1996 | 9,368,740 | 2,115,100 | 577,640 | 12,061,480 | 14,338,670 | 50,817 |
| 1997 | 9,563,190 | 2,078,900 | 644,890 | 12,286,980 | 14,275,400 | 50,288 |
| 1998 | 9,803,790 | 2,078,300 | 670,110 | 12,552,200 | 13,913,210 | 49,947 |
| 1999 | 9,705,320 | 2,044,280 | 548,640 | 12,298,240 | 10,938,170 | 49,884 |
| 2000 | 9,186,970 | 2,029,330 | 558,100 | 11,774,400 | 14,296,750 | 49,544 |
| 2001 | 8,095,880 | 1,903,600 | 582,210 | 10,581,690 | 16,094,330 | 49,719 |
| 2002 | 8,063,187 | 1,934,678 | 526,276 | 10,524,141 | 15,544,135 | 50,144 |
| 2003 | 8,006,336 | 1,938,933 | 488,256 | 10,433,525 | 14,470,923 | 50,322 |
| 2004 | 7,982,297 | 1,949,725 | 469,704 | 10,401,726 | 15,863,815 | 50,761 |
| 2005 | 7,883,060 | 1,958,882 | 469,012 | 10,310,954 | 14,704,067 | 51,094 |
| 2006 | 7,627,926 | 1,933,874 | 463,725 | 10,025,525 | 13,599,792 | 51,312 |
| 2007 | 7,498,217 | 1,898,538 | 456,669 | 9,853,424 | 14,128,954 | 51,319 |
