# Supplementary material for: A Review of Exotic Animal Disease in Great Britain and in Scotland Specifically between 1938 and 2007
Source: PLoS One. 2011 Jul 27;6(7):e22066. doi: 10.1371/journal.pone.0022066 (PMC3144883; doi:10.1371/journal.pone.0022066)
Supplement: Table S3 — Reviewed disease with maximal incidence on agricultural holdings of Great Britain each year 1938–2007. (DOC) [file pone.0022066.s003.doc]

**Table S3.** Reviewed disease with maximal incidence on agricultural holdings of Great Britain each year 1938-2007.

Data limitations: The number of holdings affected by HPAI in 1959 was not available. Numbers of holdings affected by NDV were included in the reports of British governmental veterinary services from 1947, and the numbers of new breakdowns of bTB were included from 1948.

| year | disease with maximal incidence | annual incidence, % | data on bTB available |
| --- | --- | --- | --- |
| 1938 | CSF | 0.21% | No |
| 1939 | CSF | 0.71% | No |
| 1940 | CSF | 1.09% | No |
| 1941 | CSF | 0.24% | No |
| 1942 | FMD | 0.15% | No |
| 1943 | CSF | 0.12% | No |
| 1944 | CSF | 0.32% | No |
| 1945 | CSF | 0.20% | No |
| 1946 | CSF | 0.08% | No |
| 1947 | NDV | 0.49% | No |
| 1948 | NDV | 0.06% | No |
| 1949 | NDV | 0.13% | No |
| 1950 | CSF | 0.10% | No |
| 1951 | CSF | 0.30% | No |
| 1952 | Anthrax | 0.27% | No |
| 1953 | CSF | 0.62% | No |
| 1954 | CSF | 0.33% | No |
| 1955 | CSF | 0.32% | No |
| 1956 | Anthrax | 0.29% | No |
| 1957 | NDV | 0.24% | No |
| 1958 | CSF | 0.30% | No |
| 1959 | NDV | 0.50% | No |
| 1960 | NDV | 0.57% | No |
| 1961 | Bovine TB | 1.69% | Yes |
| 1962 | Bovine TB | 1.17% | Yes |
| 1963 | Bovine TB | 0.68% | Yes |
| 1964 | NDV | 0.60% | Yes |
| 1965 | Anthrax | 0.15% | No |
| 1966 | Bovine TB | 0.43% | Yes |
| 1967 | FMD | 0.67% | Yes |
| 1968 | Bovine TB | 0.33% | Yes |
| 1969 | Bovine TB | 0.37% | Yes |
| 1970 | NDV | 1.16% | No |
| 1971 | NDV | 1.50% | No |
| 1972 | NDV | 0.14% | No |
| 1973 | SVD | 0.05% | No |
| 1974 | SVD | 0.07% | No |
| 1975 | SVD | 0.02% | No |
| 1976 | Anthrax | 0.02% | No |
| 1977 | Anthrax | 0.05% | No |
| 1978 | Anthrax | 0.06% | No |
| 1979 | SVD | 0.02% | No |
| 1980 | SVD | 0.02% | No |
| 1981 | Anthrax | 0.01% | No |
| 1982 | Aujeszky's disease | 0.02% | No |
| 1983 | Aujeszky's disease | 0.18% | No |
| 1984 | Bovine TB | 0.04% | Yes |
| 1985 | Bovine TB | 0.03% | Yes |
| 1986 | Bovine TB | 0.03% | Yes |
| 1987 | Bovine TB | 0.04% | Yes |
| 1988 | Bovine TB | 0.05% | Yes |
| 1989 | Bovine TB | 0.06% | Yes |
| 1990 | Bovine TB | 0.07% | Yes |
| 1991 | Bovine TB | 0.07% | Yes |
| 1992 | Bovine TB | 0.07% | Yes |
| 1993 | Bovine TB | 0.10% | Yes |
| 1994 | Bovine TB | 0.13% | Yes |
| 1995 | Anthrax | 0.0004% | No |
| 1996 | Bovine TB | 0.0012% | No |
| 1997 | Bovine TB | 0.20% | Yes |
| 1998 | Bovine TB | 0.29% | Yes |
| 1999 | Bovine TB | 0.34% | Yes |
| 2000 | Bovine TB | 0.37% | Yes |
| 2001 | FMD | 0.77% | Yes |
| 2002 | Bovine TB | 0.65% | Yes |
| 2003 | Bovine TB | 0.58% | Yes |
| 2004 | Bovine TB | 0.61% | Yes |
| 2005 | Bovine TB | 0.71% | Yes |
| 2006 | Bovine TB | 0.71% | Yes |
| 2007 | Bovine TB | 0.74% | Yes |
