# Supplementary material for: A Review of Exotic Animal Disease in Great Britain and in Scotland Specifically between 1938 and 2007
Source: PLoS One. 2011 Jul 27;6(7):e22066. doi: 10.1371/journal.pone.0022066 (PMC3144883; doi:10.1371/journal.pone.0022066)
Supplement: Table S5 — Livestock disease reviewed due to which the maximal percentage of susceptible animals farmed in Great Britain was culled each year 1938–2007. (DOC) [file pone.0022066.s005.doc]

**Table S5.** Livestock disease reviewed due to which the maximal percentage of susceptible animals farmed in Great Britain was culled each year 1938-2007.

Species counted as susceptible: pigs for Aujeszky's disease, CSF and SVD; cattle for bTB; cattle, sheep and pigs for anthrax and FMD.

Data limitations: The numbers culled due to Aujeszky's disease were not available for 4 of the 11 years that the disease was reported between 1938 and 2007: 1979-1982.

| year | disease with maximal cull of livestock | maximal cull of livestock, % |
| --- | --- | --- |
| 1938 | FMD | 0.06% |
| 1939 | CSF | 0.16% |
| 1940 | CSF | 0.19% |
| 1941 | FMD | 0.09% |
| 1942 | FMD | 0.19% |
| 1943 | CSF | 0.07% |
| 1944 | CSF | 0.13% |
| 1945 | CSF | 0.07% |
| 1946 | CSF | 0.03% |
| 1947 | FMD | 0.04% |
| 1948 | Bovine TB | 0.07% |
| 1949 | Bovine TB | 0.06% |
| 1950 | Bovine TB | 0.05% |
| 1951 | FMD | 0.04% |
| 1952 | FMD | 0.22% |
| 1953 | Bovine TB | 0.04% |
| 1954 | Bovine TB | 0.08% |
| 1955 | Bovine TB | 0.12% |
| 1956 | Bovine TB | 0.09% |
| 1957 | Bovine TB | 0.12% |
| 1958 | CSF | 3.00% |
| 1959 | CSF | 2.17% |
| 1960 | CSF | 2.79% |
| 1961 | CSF | 3.54% |
| 1962 | CSF | 6.34% |
| 1963 | CSF | 4.49% |
| 1964 | CSF | 1.78% |
| 1965 | CSF | 0.63% |
| 1966 | CSF | 0.13% |
| 1967 | FMD | 0.90% |
| 1968 | FMD | 0.08% |
| 1969 | Bovine TB | 0.03% |
| 1970 | Bovine TB | 0.02% |
| 1971 | Bovine TB | 0.02% |
| 1972 | SVD | 0.05% |
| 1973 | SVD | 1.10% |
| 1974 | SVD | 1.16% |
| 1975 | SVD | 0.35% |
| 1976 | SVD | 0.03% |
| 1977 | SVD | 0.12% |
| 1978 | Bovine TB | 0.01% |
| 1979 | SVD | 0.61% |
| 1980 | SVD | 0.66% |
| 1981 | SVD | 0.09% |
| 1982 | SVD | 0.14% |
| 1983 | Aujeszky's disease | 4.86% |
| 1984 | Aujeszky's disease | 0.76% |
| 1985 | Aujeszky's disease | 0.09% |
| 1986 | CSF | 0.11% |
| 1987 | CSF | 0.05% |
| 1988 | Aujeszky's disease | 0.07% |
| 1989 | Aujeszky's disease | 0.02% |
| 1990 | Bovine TB | 0.01% |
| 1991 | Bovine TB | 0.01% |
| 1992 | Bovine TB | 0.02% |
| 1993 | Bovine TB | 0.02% |
| 1994 | Bovine TB | 0.03% |
| 1995 | Bovine TB | 0.03% |
| 1996 | Bovine TB | 0.04% |
| 1997 | Bovine TB | 0.04% |
| 1998 | Bovine TB | 0.06% |
| 1999 | Bovine TB | 0.03% |
| 2000 | CSF | 1.23% |
| 2001 | FMD | 8.38% |
| 2002 | Bovine TB | 0.27% |
| 2003 | Bovine TB | 0.26% |
| 2004 | Bovine TB | 0.25% |
| 2005 | Bovine TB | 0.26% |
| 2006 | Bovine TB | 0.26% |
| 2007 | Bovine TB | 0.27% |
