# Supplementary material for: A Review of Exotic Animal Disease in Great Britain and in Scotland Specifically between 1938 and 2007
Source: PLoS One. 2011 Jul 27;6(7):e22066. doi: 10.1371/journal.pone.0022066 (PMC3144883; doi:10.1371/journal.pone.0022066)
Supplement: Table S6 — Total percentage of cattle, sheep and pigs farmed in Great Britain culled in control of the reviewed diseases each year 1938–2007. (DOC) [file pone.0022066.s006.doc]

**Table S6.** Total percentage of cattle, sheep and pigs farmed in Great Britain culled in control of the reviewed diseases each year 1938-2007.

Data limitations: The numbers culled due to Aujeszky's disease were not available for 4 of the 11 years that the disease was reported between 1938 and 2007: 1979-1982.

| year | total cull of cattle, sheep and pigs farmed, % |
| --- | --- |
| 1938 | 0.13% |
| 1939 | 0.19% |
| 1940 | 0.24% |
| 1941 | 0.15% |
| 1942 | 0.24% |
| 1943 | 0.09% |
| 1944 | 0.18% |
| 1945 | 0.11% |
| 1946 | 0.05% |
| 1947 | 0.05% |
| 1948 | 0.09% |
| 1949 | 0.08% |
| 1950 | 0.08% |
| 1951 | 0.12% |
| 1952 | 0.27% |
| 1953 | 0.08% |
| 1954 | 0.09% |
| 1955 | 0.14% |
| 1956 | 0.17% |
| 1957 | 0.20% |
| 1958 | 3.28% |
| 1959 | 2.44% |
| 1960 | 3.17% |
| 1961 | 3.74% |
| 1962 | 6.44% |
| 1963 | 4.55% |
| 1964 | 1.84% |
| 1965 | 0.67% |
| 1966 | 0.26% |
| 1967 | 0.93% |
| 1968 | 0.10% |
| 1969 | 0.03% |
| 1970 | 0.02% |
| 1971 | 0.02% |
| 1972 | 0.07% |
| 1973 | 1.12% |
| 1974 | 1.17% |
| 1975 | 0.36% |
| 1976 | 0.04% |
| 1977 | 0.12% |
| 1978 | 0.01% |
| 1979 | 0.62% |
| 1980 | 0.67% |
| 1981 | 0.10% |
| 1982 | 0.15% |
| 1983 | 4.86% |
| 1984 | 0.76% |
| 1985 | 0.10% |
| 1986 | 0.12% |
| 1987 | 0.10% |
| 1988 | 0.08% |
| 1989 | 0.03% |
| 1990 | 0.01% |
| 1991 | 0.01% |
| 1992 | 0.02% |
| 1993 | 0.02% |
| 1994 | 0.03% |
| 1995 | 0.03% |
| 1996 | 0.04% |
| 1997 | 0.04% |
| 1998 | 0.06% |
| 1999 | 0.03% |
| 2000 | 1.31% |
| 2001 | 8.45% |
| 2002 | 0.27% |
| 2003 | 0.26% |
| 2004 | 0.25% |
| 2005 | 0.26% |
| 2006 | 0.26% |
| 2007 | 0.27% |
