# Supplementary material for: A Review of Exotic Animal Disease in Great Britain and in Scotland Specifically between 1938 and 2007
Source: PLoS One. 2011 Jul 27;6(7):e22066. doi: 10.1371/journal.pone.0022066 (PMC3144883; doi:10.1371/journal.pone.0022066)
Supplement: Table S7 — The month of index case and the locality of affected holdings in Scotland. (DOC) [file pone.0022066.s007.doc]

**Table S7.** The month of index case and the locality of affected holdings in Scotland^a^.

| disease | number of years disease reported 1938-2007 | number of years month of index case reported | month (number of episodes) | number of years locality reported | locality (number of episodes) |
| --- | --- | --- | --- | --- | --- |
| anthrax | 43 | 0 | - | 4 | Central Scotland (1), Dumfries and Galloway (1), Highlands (1), Strathclyde (1) |
| FMD | 12 | 5 | Mar (2), Apr (1), May (1), Nov (1) | 6 | Aberdeen (2), Lockerbie (1), Paisley and Campbelltown (1), near river Forth (1), Turriff (1) |
| CSF | 25 | 2 | Jan (1), Jun (1) | 0 | - |
| NDV | 20 | 6 | Mar (1), May (1), Oct (1), Sept (2), Dec (1) | 12 | Aberdeenshire and Ayrshire (2), Aberdeenshire (1), Dumfries (1), East Lothian (1), Kirkcudbrightshire (1), Lanarkshire (1), Midlothian (1), Scottish Islands and Borders (1), Stranraer (1), Tayside (1), Western Islands (1) |
| SVD | 2 | 0 | - | 0 | - |

^a^Aujeszky's disease and HPAI in farmed poultry were not reported in Scotland 1938-2007. Bovine tuberculosis is not included because the numbers of holdings affected in Scotland were available inconsistently.
