# Supplementary material for: A Review of Exotic Animal Disease in Great Britain and in Scotland Specifically between 1938 and 2007
Source: PLoS One. 2011 Jul 27;6(7):e22066. doi: 10.1371/journal.pone.0022066 (PMC3144883; doi:10.1371/journal.pone.0022066)
Supplement: Text S1 — Conduct of the systematic review. (DOC) [file pone.0022066.s008.doc]

**Text S1. Conduct of the systematic review**

The systematic review of disease occurrence was conducted in accordance with recommendations of the Preferred Reporting Items for Systematic Reviews and Meta-Analyses (PRISMA) statement [7].

Objective. To review the occurrence of diseases of the former OIE List A, Aujeszky's disease, anthrax and bovine tuberculosis (bTB) in Great Britain (GB) and in Scotland specifically, from 1938 to 2007, as reported by British governmental veterinary services or other sources of similar reliability.

*Data sources:* The primary data source was the reports of British governmental veterinary services, format of which had changed over the years [6]. Following World War II, a single edition Report of Proceedings under the Disease of Animals Acts for the years 1938 to 1947 was compiled. Reports on Animal Health Services in Great Britain were produced from 1948 to 1970. Annual Report of Chief Veterinary Officer: Animal Health was published from 1971 to 2007. The number of holdings affected by each disease in GB each year indicated in the veterinary services’ reports was cross-checked against the outbreak statistics published on-line by the UK Government's Department for Environment, Food and Rural Affairs (DEFRA) (except for bTB where the latter was available only since 1998), and from 1996 onwards also against the OIE HANDISTATUS published on-line.

Several inquiries into the epidemiology of bTB in GB were commissioned during the review period; the inquiries’ reports were considered as an information source. However, it was concluded that, for consistency, only the bTB statistics from the British governmental veterinary services’ reports would be used.

Contagious diseases of farmed fish were considered, but were not reviewed because a reliable data-source of historical information was not identified.

*Data extraction:* A searchable database was compiled in Microsoft Office Access ®2003 using a data extraction form developed for this purpose. For each year that a given disease was reported in GB, the form required to entry the following upon availability: total numbers of holdings affected and animals culled due to the disease in GB (as a whole) and in Scotland alone, mode(s) of the disease's introduction into GB and into Scotland, mode(s) of the disease's spread, animal species with which the disease was introduced and spread, all animal species affected, localities of index holdings and of all the holdings affected, and number of index holdings. The duration of outbreaks was not relevant because some of the diseases reviewed were endemic in GB during a part of the reviewed period.

## *Data synthesis:* The database was exported into and analyzed in Microsoft Office Excel ®2003, where the disease statistics were calculated. Additional information on the denominator farm-animal populations was acquired and is described in *Supporting Information 2*. The figures were produced in the *R* software environment.
