# Supplementary material for: A Review of Exotic Animal Disease in Great Britain and in Scotland Specifically between 1938 and 2007
Source: PLoS One. 2011 Jul 27;6(7):e22066. doi: 10.1371/journal.pone.0022066 (PMC3144883; doi:10.1371/journal.pone.0022066)
Supplement: Text S2 — Data sources, availability and limitations. (DOC) [file pone.0022066.s009.doc]

**Text S2. Data sources, availability and limitations**

**Data on disease occurrence**

**Great Britain**

i) The test and slaughter programme for bTB in cattle became compulsory in GB in 1950 [14]. The numbers of herd breakdowns with bTB in GB have appeared in the reports of British governmental veterinary services since 1948. However these numbers were not available in the reports for 30 of the 60 years from 1948 to 2007 (Figure 2, bottom panel).

(ii) The numbers of Newcastle disease (NDV) outbreaks have appeared in the reports of British governmental veterinary services since 1947.

(iii) The numbers of holdings affected by anthrax in GB were available in all 62 years that the disease was reported in British farm-animals between 1938 and 2007; for Aujeszky's disease in all 11 years reported; for classical swine fever (CSF) in all 33 years; for foot and mouth (FMD) in all 32 years; for NDV in all 36 years; and for swine vesicular disease (SVD) in all 10 years. For highly pathogenic avian influenza (HPAI) the data were not available for one (1959) of the 5 years reported.

(iv) The numbers of animals culled in GB due to anthrax were available in all 62 years that the disease was reported between 1938 and 2007; for bTB in all 60 years post-1948; for FMD in all but one (2007) of 32 years reported; for CSF in all 33 years; and for SVD in all 10 years. The numbers of poultry culled in GB due to NDV were not available for 8 of 36 years reported: 1965, 1966, 1972-1976 and 2006. The numbers of poultry culled due to HPAI were not available for 3 of 5 years reported: 1963, 2006 and 2007. The numbers of animals culled due to Aujeszky's disease were not available for 4 of 11 years reported: 1979-1982.

(v) The locality of holdings affected by HPAI was available in all 5 years that the disease was reported in poultry in GB between 1938 and 2007. The locality of holdings with anthrax was available in only 5 of 62 years reported; for Aujeszky's disease in 2 of 11 years; for CSF in 6 of 33 years; for FMD in 21 of 32 years; for NDV in 5 of 36 years; and for SVD in 3 of 10 years. These results are not presented.

vi) The months and numbers of index cases, and the animal species with which the diseases were introduced in GB were available only in some cases, inadequately to allow a meaningful characterization throughout the 70 years reviewed. These results are not presented.

**Scotland**

(i) The numbers of outbreaks in Scotland were available for CSF in all 25 years that the disease was reported between 1938 and 2007; for FMD in all 12 years; for anthrax in all 43 years; and for SVD in both years the disease was present. For NDV the number of outbreaks in 1948 was not available (*n*=267 in GB) but was available for the other 19 years reported. The numbers of new breakdowns of bTB were consistently available only for the years 1997-2007; the disease was reported in Scotland in at least 33 of the 60 years 1948-2007. The locations reported for the outbreaks of HPAI in poultry in GB between 1938 and 2007 were outside Scotland.

(ii) The numbers of animals culled in Scotland due to anthrax were only available in 1993 and 1997; such numbers were not available for CSF or SVD in any of the years reported; for NDV they were available only in 1969; and for FMD they were available in 4 of 12 years reported. The numbers of animals culled in control of bTB in Scotland were available in 25 of the 60 years 1948-2007. Because of these data limitations, the percentages of susceptible farm-animals culled in control of the diseases reviewed in Scotland were not evaluated.

**Data on denominator populations**

## Numbers of agricultural holdings and animals farmed

## Between 1938 and 2007, a census of agriculture and horticulture was conducted in each of the three GB countries every June. The digitized census results were obtained from several sources as detailed below. There are general limitations to the data derived from the censuses’ results. The way the surveys were administered and their results publicized, the classifications of agricultural holdings, the coverage of different holding types in individual years, and other details changed throughout the 70 years. These changes and the limitations of the resulting data have been discussed elsewhere; in the present analysis the data were used exactly as available from the data sources listed.

The results of the Scottish censuses 1938-1978 were downloaded from the EDINA® service at the Edinburgh University Data Library. The data for 1982-2007 were extracted from on-line publications of the Scottish Government. The data for 1979-1981 were received from the Rural and Environment, Research and Analysis Directorate of the Scottish Government. The numbers of agricultural holdings and of cattle, sheep, pigs, fowls and total poultry farmed in Scotland were available for all of the 70 years reviewed.

The results of the Welsh censuses before 1997 were extracted from the Digest of Welsh Historical Statistics 1700 to 1974, and the Digest of Welsh Historical Statistics 1974 to 1996, both published on-line by the Welsh Assembly Government. Where possible, the total numbers of animals farmed were obtained by summation of the numbers for larger (so called “main”) and smaller holdings. The summaries of results of the Welsh censuses 1984-1999 were also received from DEFRA. The data for 2000-2007 were extracted from on-line publications of the Statistical Directorate of the Welsh Assembly Government, such as the STATSWALES initiative ([http://www.statswales.wales.gov.uk](http://www.statswales.wales.gov.uk/)) and from the reports “Farming Facts and Figures Wales” for the years 2004, 2007 and 2008. For Wales, the numbers of agricultural holdings were available for 69 of the 70 years reviewed, with the exception being the year 1940. The totals of cattle, sheep and pigs farmed were available for all of the 70 years. The total numbers of poultry farmed in Wales were located for 1938-1999 and 2006-2007, and the numbers of fowls alone for 2000-2004. The missing numbers of fowls and of the other poultry, and the number of agricultural holdings in 1940 were approximated by linear interpolation from the nearest available data, assuming a constant rate of change year-to-year. Then the total numbers of poultry farmed in Wales in 2000-2005 were obtained by summation of the numbers of fowls and of the other poultry.

The summaries of English census results for the years 1935, 1945, 1955, 1965, 1971-1977 and 1981-1999 were received from DEFRA. The summaries of the census results for 2000 onwards were available in DEFRA's on-line publications. The totals for England and Wales of the numbers of cattle, sheep, pigs and poultry farmed in each of the years 1939-1972 and in 1978 were extracted from the results of the Scottish agricultural censuses for those years, downloaded from the EDINA® service at the Edinburgh University Data Library. The numbers of agricultural holdings in England and Wales in 1950, 1960 and 1970 were obtained from the web-site of the initiative The Vision of Britain through Time (<http://www.visionofbritain.org.uk/index.jsp>). All the statistics for 1939 were used for 1938 as an approximation. The missing statistics for 1979 and 1980 were approximated by linear interpolation, assuming a constant rate of change year-to-year, from the available data for 1978 and 1981. The numbers of agricultural holdings in England were approximated by interpolation for some of the remaining years. Most notably, the numbers of holdings in 1938-1950 were interpolated from those in 1931 and in 1950; however, these numbers were similar: 333,753 in 1931 and 317,640 in 1950.

The total number of agricultural holdings in GB each year between 1938 and 2007 was obtained by summation of the numbers of holdings reported in England, Scotland and Wales. The numbers of cattle, sheep, pigs and total poultry farmed in GB each year were obtained similarly.

**Numbers of holdings farming individual livestock species or poultry**

The numbers of holdings farming individual species were not available as consistently as the other denominator data discussed above.

The numbers of holdings farming cattle and of holdings farming sheep in Scotland each year from 1966 to 1978, farming pigs from 1965 to 1978, and farming each of these three species or poultry in 2000 to 2007 were extracted from the results of the Scottish agricultural censuses downloaded from the EDINA® service at the Edinburgh University Data Library.

The numbers of holdings farming cattle, sheep, pigs, poultry or mixed livestock in Wales were available for 1991-2004. The holdings specified as farming mixed livestock were disregarded. The number of holdings with pigs and poultry in a given year was used as both the number of holdings with pigs and the number of holdings with poultry.

The numbers of holdings farming cattle, sheep, pigs or poultry (obtained as the sum of holdings farming fowls and ducks) in England in 1981-1989 were extracted from the summaries of English agricultural censuses supplied by DEFRA. In those years, ducks and geese might have been housed on the mixed-poultry holdings with fowls; therefore, the total number of poultry holdings might be overestimated. The numbers of holdings farming turkeys were available too scarcely to be used. The numbers of holdings farming cattle, sheep, pigs or poultry (the sum of holdings farming fowls and ducks) in England in 2000-2007 were obtained from the summaries of the census results for 2000 onwards available in DEFRA on-line publications.

From these data, the numbers of holdings farming cattle, sheep, pigs or poultry in each of the three GB countries throughout the 70 years were approximated. This approximation assumed that the number of holdings of each type changed throughout the 70 years in line with the trends in the total number of agricultural holdings in the country. *I.e.* the rate of change in the number of holdings of each type between any two consecutive years was taken to be exactly the same as the net rate of change in the total number of agricultural holdings in the country. The information available was insufficient to account for individual trends in the numbers of holdings of different types. However, more precise estimates were unobtainable. The number of holdings of each type was approximated as detailed above, except for two instances where the available data allowed for a linear interpolation: between 1989 and 2000 for holdings of all the types in England, and between 1978 and 2000 for holdings farming cattle, sheep or pigs in Scotland. The total number of holdings of each type in GB in each year 1938-2007 was obtained by summation of the numbers of holdings in England, Scotland and Wales. These totals were used to evaluate the annual incidence of bTB on GB cattle holdings, the annual incidences of swine exotics on GB pig holdings, and the annual incidence of NDV on GB poultry holdings.

Only the denominator of the total number of agricultural holdings in GB was used for evaluating the incidence of FMD and anthrax. This is because no adequate information throughout the 70 years was available to describe what fraction of holdings farming sheep also farmed cattle or pigs, and vice versa.
